# Acknowledgment to Reviewers of *Animals* in 2020

**DOI:** 10.3390/ani11020313

**Published:** 2021-01-27

**Authors:** Animals Editorial Office

**Affiliations:** MDPI AG, St. Alban-Anlage 66, 4052 Basel, Switzerland

Peer review is the driving force of journal development, and reviewers are gatekeepers who ensure that *Animals* maintains its standards for the high quality of its published papers. Thanks to the cooperation of our reviewers, in 2020, the median time to first decision was 17 days and the median time to publication was 37 days. The editors would like to express their sincere gratitude to the following reviewers for their precious time and dedication, regardless of whether the papers were finally published:
Abbate, Jessica MariaLi, JiaqiAbd El-Hack, Mohamed E.Li, JinquanAbdallah, Mohamed F.Li, JinyaoAbdennebi-Najar, LatifaLi, KuiAbdollahi-Arpanahi, RostamLi, QiulingAbelson, KlasLi, ShijunAbeni, FabioLi, ShucongAblondi, MichelaLi, SufenAbo-Ismail, Mohammed K.Li, Tie-JunAbramo, FrancescaLi, WeifenAbuelo, ÁngelLi, WenliAbugomaa, AmiraLi, Xian-ZhiAbu-Niaaj, LubnaLi, XiaoyuAccomando, Alyssa W.Li, XinweiAccorsi, Pier AttilioLi, XuwenAchaaban, Mohamed RachidLi, YangAcharya, ArpanLi, YihangAckerman, MatthewLi, YuzhiAcutis, Pier LuigiLi, ZhongqiuAdamczak, LechLi, ZhuanjianAdamczyk, KrzysztofLian, ZhengxingAdamiak, ZbigniewLiang, JunAdamík, PeterLiao, Xin DiAdams, SeiduLibera, JustynaAdamski, MarekLicka, Theresia F.Addie, DianeLicon-Cano, CarmenAddis, MargheritaLiedtke, Katarzyna G.Addis, Maria FilippaLiermann, WendyAdenuga, Henry AdewaleLiesegang, AnnetteAdewole, DeborahLiew, Thor-SengAdhikari, BishnuLigaszewski, MaciejAdler, SteffenLim, Han KyuAdolphs, JulianLim, Kyu-SangAdrian-Kalchhauser, IreneLima Rodrigues, Jhennyfer AlineAdur, MalavikaLima, Fábio S.Aftab, UsamaLin, Chao-NanAgerholm, Jørgen SLin, Chen-SiAgga, Getahun E.Lin, HaiAggeli, Ioanna-KaterinaLin, Hui-ChuAgnew, Dalen W.Lin, Muh-ShiÅgren, Estelle C. C.Lin, TaoAguayo, LorenaLin, XialuAgui, TakashiLin, Yi-TingAguiar, Daniel M.Lind, VibekeÁguila, LuisLindholm, AmandaAgut Giménez, AmaliaLindholm, ChristinaAhmed, Ahmed A.Lindinger, MichaelAiyegoro, Olayinka A.Lindner, Arno E.Akanno, EverestusLindsay, AngusÁkos, Maróti-AgótsLinhart, OtomarAkter, YeasminLiordos, VasiliosAlabiso, MarcoLiotta, LuigiAlagwany, MahmoudLis, MarcinAland, AndresLisandru, CapaiAlarcón López, Francisco JavierLisiecka, UrszulaAlarcon-Rojo, Alma D.Liu, BangAlbano, MarcoLiu, Cheuk LunAlbarella, SaraLiu, DanAlberghina, DanielaLiu, Guo HuaAlbéric, MarieLiu, HaiboAlbertini, MariangelaLiu, Hao-KunAlberto Díaz-Sánchez, AdrianLiu, HongbingAlbrecht, ElkeLiu, HongyunAlbright, Julia D.Liu, JianzhuAlegre, BeatrixLiu, JinxinAlegria-Moran, RaulLiu, JundiAlex, Charles E.Liu, MengyangAlexander, Brenda M.Liu, ShiminAlexander, James E.Liu, ShuaiqiAlexandre, FellousLiu, WanshengAlfson, KendraLiu, Xian-YongAlhamada, MoutazLiu, XiaoleiAli, AliLiu, YipingAli, Muhammad MuddassirLiu, Zhong-huaAlibhai, Hatim I.K.Lizhi, ZhouAlkhtib, AshrafLjubojevic, DraganaAllaoui, AbdelmounaaïmLlavanera, MarcAllard, StephanieLlonch, PolAllen, AndrewLloret, JosepAllen, BenjaminLloyd, PamelaAllen, DanielLo Verde, GabriellaAllen, MichaelŁobacz, AdrianaAllen, W.R. TwinkLobel, Phillip S.Alleva, EnricoLockwood, RandallAl-Mamun, Hawlader AbdullahLodde, ValentinaAl-Marashdeh, OmarLokhorst, KeesAl-Marzooqi, WaleedLombard, JasonAlmeida, DanielaLombardi, PietroAlmond, GlenLombardo, LucaAl-Nakkash, LaylaLomillos PÉREZ, Juan ManuelAlonge, SalvatoreLoncoman, CarlosAlonso, EvaLondono, BerlinAlonso, Marta E.Long, NathanAlonso, Marta LópezLong, PanAl-Sagheer, AdhamLongone, PatriziaAlstrup Olsen, Aage KristianLonzarich, ValentinaAlvarenga, MarcoLooper, Michael L.Alvarez, JavierLoor, Juan J.Alvarez, MarikisLopes, GraçaÁlvarez-González, Carlos AlfonsoLopes, Jordana S.Alvarez-Hess, PabloLopes, Luciano BastosAlvarez-Narvaez, SonsirayLopes, Ricardo JorgeÁlvarez-Rodríguez, JavierLopes-Marques, MónicaAlvarez-Rodriguez, ManuelLópez Albors, OctavioAlves, João CarlosLópez Lluch, GuillermoAlves, Luís Francisco AngeliLopez Seijas, JacoboAlves, Susana P.López, Bruno DíazAmado, FranciscoLópez, Miguel ÁngelAmaral, AlexandraLópez, SecundinoAmaral, Andreia J.Lopez-Bote, ClementeAmaral, Karina Bohrer DoLópez-Cepero Borrego, JavierAmasheh, SalahLópez-Cortegano, EugenioAmato, MarioLópez-Gatius, FernandoAmici, AndreaLopreiato, VincenzoAmmar Dilawar, MuhammadŁopucki, RafałAmori, GiovanniLorella, ManiscalcoAmorim, IrinaLothian, Angus J.Amorim, Renee LauferLoudon, Kate M.W.Amos, WilliamLourenco, Jeferson M.Ampuero, SilviaLouton, HelenAn, LilongLoux, Shavahn C.Anastasio, AnielloLovatt, FionaAnaya-Calvo, GabrielLove, LydiaAncillotto, LeonardoLovercamp, KyleAnderson, KenLu, Hong S.Andersson, RobbyLu, HongxuAndoh, TadashiLu, Ming-WeiAndonov, AntonLu, MingzhouAndrade-Montemayor, Héctor MarioLucchese, LauraAndrás, GáspárdyLucci, CarolinaAndre, MarcosLuciana, RossiAndré, Marcos RogérioLucini, CarlaAndrea, IanniLucke, AnnegretAndreani, GiuliaŁuczyńska, JoannaAndreani, Nadia AndreaLuddi, AliceAndré-Fontaine, GenevieveLudwiczak, AgnieszkaAndren, HenrikLudwig, ArneAndreoni, ValentinaLugar, DrewAndrés, SoniaLuis Pereira, GuilhermeAndrighetto, IginoLukasik, KarolinaAndrysic, ZdenekLukaszewicz, Ewa TeresaAngel, RoselinaŁukaszewicz, MarekAngelici, Francesco M.Lunesu, MondinaAngelici, Maria CristinaLunge, Vagner RicardoAngelini, CorradoLuo, ChenglongAngelopoulou, KaterinaLuo, DongwenAngeoletto, Fábio Henrique SoaresLuo, HailingAnge-van Heugten, KimberlyLupatsch, IngridAnglani, FrancaLupo, Karen D.Angrecka, SabinaLurette, AmandineAnifandis, GeorgeLürzel, StephanieAnis, EmanLutnicki, KrzysztofAnna, Carabus FloresLuttermann, ChristineAnna, Lange-ConsiglioLutz, CorrineAnnandale, HenryLymperopoulos, AristotelisAnnicchiarico, GiovanniLynch, EmilyAnnunziata, GiuseppeLynegaard, Julie C.Antanaitis, RamunasLyons, RussellAntoniassi, RosemarLyons, TamsinAntonopoulou, EfthimiaMa, WenqiangAntunes, LuisMa, YibaoAntuofermo, ElisabettaMa, YongxiAo, TuoyingMa, YuehuiAparicio, Miguel A.Ma, YuzhongAppelbaum, SamuelMaak, SteffenApperson, Charles S.Mabjeesh, Sameer J.Aquilani, ChiaraMacdonald, DavidArango Duque, GuillermoMacedo Barragán, Rafael JulioAraújo, Lúcio FrancelinoMacGregor-Fors, IanArbieu, UgoMachado, Gisele FabrinoArcher, Gregory S.Machado, Rosangela Z.Archer, JoyMachado, ViniciusArchibald, AlanMachen, RichardArczewska-Włosek, AnnaMacias Cruz, UlisesArevalo, EsterMacLaren, Leslie A.Argente, María-JoséMacLeod, IonaArhant, ChristineMacNamara, MaureenArias Sánchez, RamónMacNeill, AmyArias, Rodrigo E.Macphail, Catriona M.Aris, AnnaMadder, MaximeAritaki, MasatoMadeira, Marta S.Ariyarathna, HarshaMademtzoglou, DespoinaArkow, PhilMadkour, AichaArmani, AndreaMadsen, Johannes GulmannArmengol, RamonMaeda, TeruoArmero Ibáñez, EvaMaertens, LucArnal, Mari CruzMagalhães-Sant’Ana, ManuelArnold, JanoschMagallanes, SergioArnott, GarethMagariños Ferro, BeatrizArranz Santos, Juan-JoseMaggi, Ricardo G.Arrazola, AitorMaggiolino, AristideArriagada, GloriaMagi, Gian EnricoArriaga-Jordan, CarlosMahmoud, AymanArsenault, JulieMahony, Timothy JohnArsenos, GeorgiosMaia, MargaridaArsi, KomalaMaier, GabrieleArtur Nishioka, RombensoMaiorano, GiuseppeArvidsson-Segerkvist, KatarinaMaiorka, AlexAsada, MasahitoMajecka, KatarzynaAsakawa, MitsuhikoMakagon, Maja M.Asher, GeoffMakecha, RadhikaAshry, MohamedMakovický, PavolAsnaghi, ValentinaMakowiecki, DanielAssmann, Tangriani SimioniMakowska, JoannaAstiazaran-Garcia, HumbertoMalacarne, MassimoAstiz, SusanaMalavasi, RacheleAtaollahi, ForoughMalbon, AlexandraAtes, SerkanMalchow, JuliaAthanasiou, Labrini V.Maldonado, JuanAthorn, RebeccaMalheiros, RamonAthrey, GiridharMalli Mohan, Ganesh BabuAtique, UsmanMalmkvist, JensAttia, YoussefMałopolska, MartynaAttili, Anna RitaMaltez, LucasAtwill, Edward R.Maltz, EphraimAtwood, Todd C.Mamrot, JaredAtxaerandio, RaquelMamuris, ZissisAuliya, MarkManafiazar, GhaderAumiller, TobiasManchester, AlisonAungier, SandraMancianti, FrancescaAurich, ChristineMancini, EmilianoAurich, JoergMancini, SimoneAvallone, LuigiMandel, RoiAvelino, ÁlvarezMandell, IraAvenant-Oldewage, AnnemariéMandrioli, LucianaAvila, Felipe FManera, MaurizioAvilés Ramírez, CarmenManfredi, JaneAviles, ManuelManfredi, Maria TeresaAwayda, MouhamedManfrin, ChiaraAwosile, BabafelaMangwe, Mancoba C.Axelaaon, ErikManiaci, GiuseppeAyala, Maria D.Manjarin, RodrigoAyalew, Lisanework EMannelli, FedericaAyuso, MiriamManriquez, DiegoAzab, WalidMans, BenAzain, MichaelMans, ChristophAzam, Aa HaerumanMansell, PeterAzarnia Tehran, DomenicoMansfield, Caroline S.Aznar, Francisco JavierManso-Díaz, GabrielBaaissa, BabelhadjMantovani, RobertoBaars, TonManu, HayfordBabatunde, KehindeManuelian, CarmenBabicz, MarekManzocchi, ElisaBabinszky, LászlóMao, DaganBacci, BarbaraMao, YongjiangBacco, ManlioMapiye, CletosBach, LydiaMarana, Moonika HaahrBachmann, MartinMarc, DanielBąchor, RemigiuszMarcato, Simara MarciaBaciadonna, LuigiMarceau, Justin F.Back, WimMarcellin-Little, Denis J.Backus, Brittany L.Marcet-Rius, MíriamBadino, PaolaMarchesini, GiorgioBadiola, IgnacioMarchetti, VeronicaBagnato, AlessandroMarchitelli, CinziaBagnicka, EmiliaMarcinčák, SlavomírBai, ShipingMarco, DaddaBaidoo, EdwardMarco, RussoBaidoo, SamMarcondes, Marcos InácioBailey, DerekMarcovici, GenoBailey, JarrodMarei, WaleedBailey, TomMarek, LukaszewiczBain, MaureenMarelli, Stefano PaoloBaird, BonnieMarenzoni, Maria LuisaBajcsy, ÁrpádMargarita , Sharipova R.Bajwa, JangiMargerison, JeanBakaloudis, DimitrisMargulis, SusanBaker, JaniceMaria, Gustavo A.Baker, LivMaria, RebordãoBakloushinskaya, IrinaMaria-Jesus, PalomoBalan, PrabhuMaribo, HanneBalasubramanian, BalamuralikrishnanMarie-Amélie, Forin-WiartBalcells, JoaquimMarín Orenga, ClaraBalčiauskas, LinasMarín, Andrés Luis MartínezBalčiauskienė, LaimaMarini, DanilaBaldi, AntonellaMarino, FabioBaldinger, LisaMariti, ChiaraBalestrieri, AlessandroMarklund, UlrikaBalocchi, OscarMarlin, DavidBalzani, AgneseMármol-Sánchez, EmilioBanaszak, MirosławMaros, KatalinBanaszewska, DorotaMaroto-Molina, FranciscoBandara, Rathnayaka Mudiyanselage Amila SubhashinieMarounek, MilanBani, LucianoMarques Allegretti, SilmaraBanias, OvidiuMarques, MarianaBao, JunMarruchella, GiuseppeBao, WenbinMarsano, René MassimilianoBaquero, Monica M.Marschang, Rachel E.Baracchi, DavidMarsh, DavidBaragli, PaoloMarshall, LindsayBaranov, Maksim V.Marsico, GiuseppeBarba Recreo, MartaMarsilio, FulvioBarba, Cecilio JoséMartelli, PaoloBarbaresi, AlbertoMarth, ChristinaBarbari, MatteoMarti, SoniaBarbas, João PedroMartín Castillo, MargaritaBarbato, MarioMartín, AdrienneBarbera, SalvatoreMartin, JessicaBarberio, AntonioMartin, KarinBarbieri, SaraMartín-Burriel, InmaculadaBarbosa, SoraiaMartín-Cuervo, MaríaBarcena, CarmenMartínez Miró, SilviaBard, Kim A.Martinez, Charles CanBardehle Parra, LeonardoMartínez, TomasBarenys, MartaMartínez, YordanBarile, Vittoria LuciaMartinez-Izquierdo, EstibalizBarja, Juan L.Martínez-López, VicenteBarker, StephenMartínez-Morcillo, Francisco J.Barker, Timothy HMartinez-Pastor, FelipeBarłowska, JoannaMartínez-Pérez, PedroBärmann, EvaMartinez-Silvestre, AlbertBarnard, ShanisMartín-García, IgnacioBarnes, Michael E.Martín-Hidalgo, DavidBaro, Jesus A.Martini, AndreaBarr, AlistairMartini, ValeriaBarragan, Adrian A.Martino, CamilloBarrasso, RobertaMartino, GiuseppeBarrio, Isabel C.Martino, Luisa DeBarros-Velázquez, JorgeMartino, Piera AnnaBarszcz, KarolinaMartins, EvaBarszcz, MarcinMartins, LuísBarthel, Leon M. F.Martins, Marta FonsecaBartlett, JannetteMartins, Maurício LaterçaBartolomé Medina, EsterMartins, Nelson Rodrigo Da SilvaBartolomé, JordiMartins-Bessa, AnaBarton, MaryMarume, UpenyuBartoš, LuděkMaselli, ValeriaBasaglia, FedericaMaśko, MałgorzataBasak, AninditaMassacci, Francesca RomanaBasdagianni, ZoitsaMastalerczuk, GrażynaBasioura, AthinaMastrangelo, SalvatoreBasiricò, LoredanaMasucci, FeliciaBąska, PiotrMatafome, PauloBassetto, César CristianoMateo, JavierBastien, CélyneMateos, CristianBatargias, CostasMateos, IvánBathgate, RoslynMatera, Julia MariaBatista, PatríciaMather, JenniferBatkowska, JustynaMathurkar, ShashwatiBatorska, MartynaMatics, ZsoltBattaglini, Luca MariaMátis, GáborBattini, MonicaMatoba, ShogoBattisti, CorradoMatsubara, HajimeBauer, BenMatsubara, RyumaBauer, ChristianMatsubayashi, MakotoBaumgartner, KatrinMatsumoto, HirokazuBaumgartner, MiriamMatthews, JamesBauquier, JenniMatthews, LindsayBaymakova, MagdalenaMattiello, SilvanaBaynes, RonaldMattos Veloso, CristinaBazylak, GrzegorzMaublanc, Marie LineBazzano, MarilenaMaurer, PatricBearman-Brown, LucyMauro, VasconiBeasley, ErinMavrogianni, Vasia S.Beasley, SheaMavrommatis, AlexandrosBeaulieu, DeniseMawson, PeterBeaver, BonnieMaxwell, HerrisBebbere, DanielaMay, Clive N.Beccati, FrancescaMayhew, JoeBeck, AlanMazancová, JanaBeckel, Jonathan M.Mazgajski, Tomasz D.Becker, DoreenMazrier, HamutalBecker, MartinMazurkiewicz, JanBed’Hom, BertrandMazur-Kuśnirek, MagdalenaBeddoe, TravisMazzamuto, Maria VittoriaBednarczyk, MarekMazzei, MaurizioBedore, Jessica SuageeMazzola, Silvia MichelaBeever, JonMazzoni, ChiaraBeggel, SebastianMazzoni, MaurizioBeggs, David SMazzuoli-Weber, GemmaBehm, JocelynMcCarthy, FionaBeiki, HamidMcCarty, Richard C.Beitz, Donald C.McCobb, EmilyBelanche, AlejandroMcCoy, AnnetteBelenguer, ÁlvaroMcCrae, PersephoneBelghit, IkramMcDaneld, Tara G.Bell, Jerold S.Mcdonald, JenniferBellagamba, FedericaMcDonald, TanyaBellezo, FaustoMcDonnell, Sue M.Belli, Carla BargiMcDuffee, Laurie A.Bello, Nicholas T.McFarlane, JamesBeltman, MarijkeMcFarlane, SamanthaBeltrán Frutos, EsterMcGill, JodiBełżecki, GrzegorzMcGovern, FionaBenato, LiviaMcGregor, HughBenazzi, CinziaMcGrew, MikeBenchaar, ChaoukiMcGuire, BettyBenedetto, MorandiMcLean, AmyBenítez, RitaMcLennan, KristaBenmerzouga, ImaanMcLeod, Emily M.Bennato, FrancescaMcLeod, LynetteBennet, EuanMcManus, ConceptaBennett, RichardMcMichael, Maureen A.Benowitz, Kyle M.McMillan, FrankBenson, EricMcMillin, Kenneth W.Benten, AnkeMcMurray, David N.Bentosela, MarianaMcParland, SinéadBenyshek, Daniel C.McQuade, RachelBenzertiha, AbdelbassetMeade, Kieran G.Beran, JanMeagher, Rebecca KathleenBerezowski, JohnMedger, KatarinaBerg, CharlotteMedica, PietroBergadano, AlessandraMedina, Félix ManuelBergdahl, AndreasMedrano, Juan HernandezBerge, Anna CatharinaMedvedev, Ilya NikolaevichBergen, WernerMeehan, CherylBerger, AnneMeier, JacquelineBerger, JeanineMeijboom, FranckBergfelt, DonMeijer, EllenBergmann, ShanaMeike, JesseBerillis, PanagiotisMeimberg, HaraldBerliner, ElizabethMeinhardt, AndreasBerman, AmielMekuchi, MiyukiBermejo-Álvarez, PabloMeléndez Suárez, DanielaBernabò, NicolaMelendez, PedroBernal-Bayard, JoaquínMelis, ClaudiaBernard, JohnMellado, MiguelBernard, RileyMelters, DanielBernstein, MarkMelzer, NinaBerrocoso, Julio Francisco DíazMenanteau-Ledouble, SimonBertelloni, FabrizioMenchetti, LauraBerteselli, Greta VeronicaMenconi, VascoBertin, Francois-ReneMendel, MartaBertini, SimoneMendonça, Renata S.Bertocchi, MartinaMendonça, TiagoBertolotti, LuigiMendoza García, Francisco JavierBertone, JoeMendyk, RobertBertoni, Giuseppe (Bern)Meneguz, MarcoBertoni, Giuseppe (Piacenza)Meng, HeBertram, MirandaMeng, ShujuanBertzbach, Luca DaniloMenna, Lucia FrancescaBęś, AgnieszkaMenozzi, AlessandroBessei, WernerMensah, Enoch A.Bester, LindaMensching, AndréBeths, ThierryMente, ElenaBette, MichaelMenzies, PaulaBettschart-Wolfensberger, RegulaMeomartino, LeonardoBeugnet, FrédéricMercati, FrancescaBevilacqua, AntonioMercer, JennyBezerra, LeilsonMerck, MelindaBezirtzoglou, EugeniaMerino, Eva Maria PerezBhak, JongMerkies, KatrinaBhattarai, SheevaMerle, RoswithaBhatti, Muhammad AzherMerlo, BarbaraBhoora, RakshaMescolini, GiuliaBiagi, GiacomoMesquita, JoãoBiagini, GiuseppeMesquita, Thássio Ricardo RibeiroBiałek, MałgorzataMetodiev, NikolaBiasato, IlariaMetrione, LaraBiasetti, PierfrancescoMetwally, MayadaBiasibetti, ElenaMetzger, JuliaBickley, StacieMetzler-Zebeli, Barbara U.Bicudo, Alvaro J. A.Meucci, ValentinaBidokhti, Mehdi RMeyer, LeithBielas, WiesławMeyer, Robert E.Bienboire Frosini, CecileMeyer-Rochow, Victor BennoBiesek, JakubMeza Herrera, César A.Biffani, StefanoMézes, MiklósBigliardi, EnricoMiar, YounesBilal, GhulamMicek, PiotrBindels, JacquesMicha, EvgeniaBionaz, MassimoMichal, FiedorowiczBir, CourtneyMichalczuk, MonikaBirch, Julie MelstedMichie, CraigBirke, Lynda I.A.Michiels, JorisBirolo, MarcoMiciński, JanBiscarini, FilippoMielcarek-Bocheńska, PaulinaBittar, Carla Maris MachadoMierzejewska, EwaBjalobok, FaithMiglio, AriannaBlache, DominiqueMigliore, GiuseppinaBlack, RandiMignon-Grasteau, SandrineBlackie, NicolaMigocka-Patrzałek, MartaBlackman, SimoneMiguel Carracha Charneca, RuiBlaha, ThomasMiguel-Pacheco, Giuliana G.Blanco, MireiaMikko, SofiaBland, IanMikuła, RobertBlank, RalfMikulski, DariuszBlanton, LucasMilani, ChiaraBlasi, DaleMilczarek, AnnaBlatchford, Richard A.Miler, KrzysztofBlavi, LaiaMiler, TriciaBlažek, RadimMilerski, MichalBleach, EmmaMilisits, GáborBlengini, CeciliaMillen, Danilo D.Blikslager, AnthonyMiller, Amy L.Block, JannaMiller, Christopher PatrickBlokhuis, HarryMiller, David S.Blum, Jason L.Miller, GemmaBlythe, Linda L.Miller, KatherineBobbo, TaniaMills, ErezBobe, GerdMilne, GeorginaBochicchio, VincenzoMin, Byengryel R.Bochniarz, MariolaMinervino, Antonio Humberto HamadBocian, AleksandraMinnee, ElenaBockstahler, BarbaraMinnelli, CristinaBogado Pascottini, OsvaldoMinor, Radiah C.Bogliolo, LuisaMinucci, SergioBögner, DesislavaMinuti, AndreaBogo, Mauricio R.Miranda, MartaBogusławska-Tryk, MonikaMiró, FranciscoBöhm, VolkerMiro, JordiBoiani, MicheleMir-Sanchis, IgnacioBöker, Kai O.Miscioscia, ErinBoldura, Oana-MariaMishina, TappeiBolwell, CharlotteMishra, BirendraBombail, VincentMishra, Santosh K.Bombardi, CristianoMiskulin, IvanBombik, ElżbietaMiśta, DorotaBonato, MaudMitchell, KatharynBonelli, FrancescaMitrus, SławomirBonfatti, ValentinaMiura, TakeshiBongalhardo, Denise CalistoMiyake, YoichiBonnet, Alejandro SuarezMiyata, YasuyoshiBonnett, Brenda N.Mizobe, FumiakiBonos, EleftheriosMizukawa, HazukiBonsembiante, FedericoMizunoya, WataruBontadina, FabioMlambo, VictorBoone, JohnMo, DelinBooth, Mark A.Moate, Peter J.Boraschi, DianaMoawad, AdelBorchardt, StefanMoberly, Heather K.Bordes, María Dolores LlobatMochizuki, HiroyukiBorges, PedroModesto, PaolaBorkovcová, MarieMoeller, Steven J.Borowski, ZbigniewMoerlein, DanielBorrelli, EmmaMogielnicka-Brzozowska, MarzenaBorrelli, LucaMogil, JeffreyBortolami, AlessioMohan-Gibbons, HeatherBorzée, AmaëlMolento, Carla Forte MaiolinoBos, BramMolina, AntonioBosch, JaimeMolina-Alcaide, EduardaBoschetti, ElisaMøller, Steen HenrikBoschiero, ClarissaMollo Neto, MarioBoselli, EmanueleMollo, AntonioBosi, PaoloMolnár, AndorBosma, RoelMolotsi, AnnelinBöswald, LindaMonchatre-Leroy, ElodieBotello-Álvarez, EnriqueMongillo, PaoloBöttger, ChristianMonk, Jessica E.Bouchnick, RamMonllor Guerra, PaulaBourneuf, EmmanuelleMontaldo, HugoBournez, LaureMonteagudo Ibañez, LuisBovera, FulviaMonteiro-Neto, ValérioBovo, SamueleMontesinos, VicenteBowen, JonMontgomery, Tracy M.Bowles, DavidMonti, GustavoBowsher, Julia H.Montoya Alonso, Jose AlbertoBoyle, LauraMoody, Carly M.Bozzi, RiccardoMoorby, JonBozzo, GiancarloMoore, KarenBragaglio, AndreaMoore-Colyer, MerielBran, José A.Moraes, GilbertoBrasca, MilenaMora-Gutierrez, AdelaBrauchle, MichaelMorais, TiagoBrauer, JulianeMorais, Tiago G.Bravo-Lamas, LeireMorales De La Nuez, AntonioBravo-Rivera, ChristianMorales, AlejandroBray, Heather J.Morales-González, José AntonioBrearley, JackieMorales-Serna, Francisco NeptalíBrehmer, AxelMorchón, RodrigoBreitbart, HaimMoreira Da Costa, LuisBremm, CarolinaMoreira, Vinicius R.Brennan, CharlesMorelli, SimoneBrereton, JamesMorello, Gabriela MunhozBresciani, CarlaMoretti, RiccardoBressan, Maria CristinaMorgan, KathleenBreton, Timothy S.Morgan, KathyBreves, GerhardMorgan, NatalieBriant, ChristineMorgante, MassimoBridges, PhillipMori, EmilianoBriefer Freymond, SabrinaMorick, DannyBriganti, AngelaMorikawa, KazuyaBriggs, BrandonMorimura, NarukiBright, TerriMorittu, Valeria MariaBrignolo, LaurieMorley, Barbara J.Brinchmann, Monica FengsrudMoroni, BarbaraBrinkløv, SigneMorris, Kevin N.Brito, Nuno V.Morris, PaulBritt, Jack H.Morris, Stephen T.Britti, DomenicoMorris, TimBroadway, Paul R.Morrison, SarahBrockmann, Gudrun A.Morris-Schaffer, KeithBrodacki, AntoniMorsy, TarekBroenstad, AuroraMortier, FemkeBronzo, ValerioMorton, DavidBrooks, RonaldMoss, AmyBrooks, Samantha A.Mostafa, AhmedBroom, Leon J.Motoie, GabrielaBrowman, HowardMott, AlexanderBrown, Christopher LyonMoula, NassimBrown, CulumMoura, Ana Silvia A. M. T.Brown, Donald J.Moura, ArlindoBrown, HiekeMoura, Marcelo TigreBrown, JanineMousel, Michelle R.Brown, JenniferMoustsen, Vivi AarestrupBrown, Wendy Y.Moya, DiegoBruce, Heather L.Moyano, FranciscoBruckner, Donald W.Moyle, Jonathan R.Brufau, JoaquimMozdziak, PaulBrugger, DanielMucchetti, GermanoBrümmer-Holder, MiekeMucha, JoannaBrunelli, ElviraMucha, MarionBrunet, FrédéricMuchacka, RenataBrunetti, GiacominaMuela, AlexanderBrunetti, LuigiMugnai, CeciliaBrunner, JesseMuhammad, TahirBruun, Thomas SønderbyMuiño, RodrigoBryan, DervanMukiibi, RobertBrys, JoannaMulero-Pázmány, MargaritaBrzozowski, MarianMuller, AnandaBu, DengpanMüller, CeciliaBuchmann, AnneMüller, KarinBuchner, FlorianMüller, NorbertBuckland, EmmaMüller, ThomasBuckley, PetraMullineaux, ElisabethBudachetri, KhemrajMunang’andu, Hetron MweembaBudik, SvenMunday, JohnBuecking, MarkMunkittrick, KellyBueno Dalto, DanyelMunoz Juzado, AnaBuhar, JeffMuñoz, AnaBuhr, Richard JeffMunoz, MariaBuijs, StephanieMuñoz-Poblete, CarlosBuitenhuis, Albert JohannesMunsterhjelm, CamillaBulc, MichałMunsters, Carolien C.B.M.Bulska, EwaMunyaka, Peris M.Bungartz, GerdMura, Maria ConsueloBungo, TakashiMurakami, HitoshiBurchfield, Keri B.Murani, EduardBurda, HynekMurawska, DariaBurdick Sanchez, NicoleMURGIANO, LeonardoBures, Regina M.Murillo, Amy C.Burghardt, GordonMurphy, SeanBurgos, RafaelMurray, PeterBurke, Joan M.Murugaiyan, JayaseelanBurkett-Cadena, Nathan D.Musch, MarkBurkina, ViktoriiaMusco, NadiaBurnett, Tracy A.Musella, VincenzoBurton, EmilyMusk, Gabrielle C.Bush, Stephen J.Muszyński, SiemowitBushby, Philip A.Muthye, VirajBussalleu, EvaMutinelli, FrancoBustamante, Hedie A.Muzio, GiulianaBustamante, RocíoMwanza, MulundaBustos-Martínez, JaimeMyers Hill, GretchenButaye, PatrickMykkänen, Anna KristinaButler, DeborahMysterud, AtleButler, GillianNääs, IrenilzaByosiere, SarahNadeau, ElisabetByrd, Christopher J.Nadler, Steven A.Byrd, James AllenNafus, MeliaCaballero, Maria JoséNagamine, YoshitakaCaballero-Villalobos, JavierNagasawa, KazueCablk, Mary E.Nagashima, Jennifer B.Cabrita, Ana Rita JordãoNagayasu, EijiCacciotto, CarlaNagel, ChristinaCadavez, VascoNaguib, MahmoudCadiergues, Marie ChristineNahashon, Samuel N.Cadogan, David J.Naidenko, SergeyCaeiro, CatiaNainiene, RasaCaffara, MonicaNair, Meera SurendranCahaner, AvigdorNair, VenugopalCai, DeminNaitana, SalvatoreCai, MinminNakajima, IkuyoCai, YafeiNakazato, GersonCai, ZexiNakazato, LucianoCaivano, DomenicoNalon, ElenaCaixeta, LucianoNanni Costa, LeonardoCajarville, CeciliaNannoni, EleonoraCakebread, PeterNapolitano, FabioCalabrò, SerenaNapolitano, FrancescoCalder, ChristineNarayan, EdwardCałka, JarosławNariaki, NonakaCallaway, Todd R.Nashta, Ali FouladiCallegari, Maria LuisaNäslund, JoacimCalsamiglia , Sergio B.Nassour, AbdallahCalver, Michael C.Natalello, AntonioCalvo, JorgeNathanailides, CosmasCamacho Vallejo, María EsperanzaNatoli, EugeniaCamacho, Jasmin J.Nava, GerardoCâmara, Diogo RibeiroNavarro Marcos, CarlosCamarda, AntonioNavarro-Martín, LaiaCambra Bort, Josep M.Navas González, Francisco JavierCamenzind, SamuelNavas-Enamorado, IgnacioCamerlink, IreneNavin, Chelliah V.Cameron, LornaNdegwa, Eunice N.Camp, Jeremy V.Neal, Mark B.Campanile, GiuseppeNeave, HeatherCampbell, DanaNeethirajan, SureshCampbell, KarlNeglia, GianlucaCampbell, MadeleineNeill, ClintonCampera, MarcoNekaris, AnneCampion, FrankNelli, RahulCampler, Magnus R.Nery, JoanaCampos, Maria Jorge GeraldesNeubauer, ViktoriaCamus, MelindaNeuditschko, MarkusCandellone, AlessiaNeuhuber, Winfried L.Cañon, JavierNeupane, MaheshCantor, Melissa C.Nevin, OwenCantos-Barreda, AnaNewcomer, Benjamin W.Cao, BinyunNewman, Shelley JoyCao, SuizhongNg, Chen SiangCao, YangchunNg, ZenithsonCao, ZhijunNguyen, TH (Huyen)Caparros-Martin, JoséNi, XueqinCapela E Silva, FernandoNicholas, FrankCapillo, GioeleNicholas, RobinCapion, NynneNichols, MargaretCapoccioni, FabrizioNiciura, SimoneCapomaccio, StefanoNickla, Debora L.Cappai, Maria GraziaNicodemus, MollyCappellesso, RoccoNicodemus, NuriaCappelli, KatiaNicol, ChristineCappello, TizianaNiczyporyk, Jowita SamantaCaprarulo, ValentinaNie, Guo-XingCapucchio, Maria TeresaNie, PinCaputo, ValerioNiedziałkowska, MagdalenaCarabaño, María JesúsNiehaus, AndrewCarbone, ChrisNielsen, BrianCarcangiu, VincenzoNielsen, Martin K.Card, ClaireNiemi, JarkkoCardoso, Clarissa SilvaNiero, GiovanniCarillo, FelicettaNiewiadomska, ZuzannaCarini, Robert M.Nii, TakahiroCarla, LuciniNikinmaa, MikkoCarluccio, AugustoNiknafs, ShahramCarneiro, PauloNiksirat, HamidCarney, Hazel C.Niku, MikaelCaroprese, MariangelaNilforooshan, Mohammad AliCarossino, MarianoNinhaus-Silveira, AlexandreCarpio, AntonioNinomiya, ShigeruCarr, MandiNishida, TakehiroCarr, NeilNishijima, KazutoshiCarragher, JohnNishijima, Ken-ichiCarregaro, Valéria LaraNishimura, NaomichiCarrera, MónicaNitulescu, MihaiCarretero, Miguel A.Niu, MutianCarretón, ElenaNiwińska, BarbaraCarrillo, José M.Nixon, Daniel W.Carrillo, WilmanNixon, JaneCarro, María DoloresNiżański, WojciechCarroll, GraceNizza, AntoninoCarter, AnneNkukwana, ThobelaCarter, JenniferNóbrega, Rafael HenriqueCaruso, GiuliaNoce, AntoniaCarvalho, António PauloNocera, IreneCarvalho, ConstançaNocillado, JosephineCasas, FabiánNogalska, AnnaCascone, GiuseppeNogalski, ZenonCasey, Theresa M.Nogueira, Daniel M.Cassandro, MartinoNogueira-Ferreira, RitaCassar, GlenNolasco-Soria, Héctor G.Cassidy, Lauren C.Nomura, KazuharuCastaldo, LucianaNonacs, PeterCastejón-Riber, CristinaNonneman, Danny J.Castellari, MassimoNora Drum, JessicaCastellaro, Giorgio G.Nordgren, LenaCastellini, CesareNordquist, RebeccaCastiglioni, BiancaNorman, TrevorCastillo Lopez, EzequiasNormando, SimonaCastillo, CristinaNorris, Jacqueline M.Castillo, PabloNorton, ElaineCastillo-Fernandez, JuanNöthling, Johan OlivietteCastorena-Torres, FabiolaNout-lomas, YvetteCastro Esteves, Pedro JoséNovák, KarelCastro, Fidel OvidioNovelli, EnricoCastro-Montoya, JoaquinNowak-Chmura, MagdalenaCaulfield, MalcolmNowakiewicz, AnetaCavalleri, Jessika-MaximilianeNowakowicz-Dębek, BożenaCavallero, SerenaNtemou, ElissavetCazenille, LeoNuccitelli, RichardCazzato, EugenioNunamaker, ElizabethCeacero, FranciscoNuñez, Cassandra M.V.Ceballos, Maria CamilaNuzzo, DomenicoCeccarelli, PieroNynca, JoannaCeccobelli, SimoneNzelu, ChukwunonsoCeciliani, FabrizioO’Brien, BernadetteCeli, PietroO’Brien, CatherineCenci-Goga, Beniamino T.O’Callaghan, Tom F.Cerasoli, IlariaO’Handley, RyanCercone, MartaO’Malley, Carly I.Cerezo, Maria Luisa MartinO’Riain, M. JustinCerini, DianaO’Shea, CormacCerino, StefaniaOanh, Nguyen CongCerolini, SilviaOatley, Jon M.Cerqueira, Aloysio M. F.Oba, EuniceCerquetella, MatteoOba, MasahitoCerri, DomenicoOber, Christopher P.Cervantes, IsabelOberg, Kerby C.Cesar De Andrade, AndréObremski, KazimierzCésar, Aline Silva MelloOchoa-Leyva, AdrianCesarani, AlbertoOchota, MałgorzataChaber, Anne-LiseOcklenburg, SebastianChagas, Juana C. CaririOczkowicz, MariaChai, JinOczkowski, MichałChai, Jong-YilOdemer, RichardChai, LilongOdoch, TerenceChalghoumi, RajaOdore, RosangelaChalmer, IainOgata, NiwakoChalvatzi, SofiaOgawa, ShinichiroChambers, PaulOgi, AsahiChami, Daniel ElOh, ChulhongChamorro, ManuelOh, Kevin P.Chan, Kin OnnOhbayashi, NorihikoChandler, Cynthia KayOhh, Sang-jipChang, Hsuan-TingOhta, MitsuakiChang, Hui-WenOhta, NaomiChang, PengxiangOhtsuka, AkiraChang, Wei-JenOikonomidis, Ioannis L.Chapman, Hilary DavidOjo, MercyChapman, Katherine E.Okafor, Chika C.Chapman, MeredithOkino, Cintia HiromiChapwanya, AspinasOláh, AttilaCharmley, EdOldenbroek, KorCharvet, ClaudeOlech, MonikaChase, LarryOlgun, OsmanChastant-Maillard, SylvieOliveira, Ronaldo LopesChaucheyras-Durand, FrederiqueOliveira-Filho, José P.Chauhan, SurinderOliver, JoAnneChaves, AlexandreOlivera, MarthaChaves, ElenaOlives, Ana Martí-DeChay-Canul, AlfonsoOlivotto, IkeChedea, VeronicaOlkowski, BogusławChen, Ching-YiOlmedo-Juárez, AgustínChen, ChongxiaoOlsen, GlennChen, GuohongOlsson, AnnaChen, HongquanOlukosi, Oluyinka A.Chen, Jiann-ChuOlynk Widmar, Nicole J.Chen, LianminOmi, ToshinoriChen, Ming-JuOnogi, AkioChen, Po-WenOnukwufor, John O.Chen, ShanyuanOnuma, ManabuChen, Shih-ChuOnzima, RobertChen, Shuen-EiOosthuizen, NicolaChen, XiaowuOpaliński, SebastianChen, Yi-FanOrczewska-Dudek, SylwiaChen, Yung-ChiaOrengo, JuanCheng, HengweiOrihuela, AgustinCheng, JianOrkusz, AgnieszkaCheng, JianboOrlowski, SaraCheng, Vincent Y-HOrnaghi, MarianaCheng, Yeong-HsiangOrr, BronwynChenoweth, PeterOrtalda, ChristianChenwen, XiaoOrtega, SofiaChetri, MadhuOrti, GuillermoCheung, Man KitOrtín Pérez, AuroraChiandetti, CinziaOrtuño, JordiChiang, Hsin-IOrusa, RiccardoChiavaccini, LudovicaOrzelska-Górka, JolantaChicharro, DeborahOsajima, Josy A.Chico Gras, VerónicaOsburn, WesleyChidgey, KirstyOsei-Amponsah, RichardChimienti, MariannaOssi, FedericoChimonyo, MichaelOstanello, FabioChincarini, MatteoOster, MichaelChistyakov, Vladimir AnatolievichOsthoff, GernotChitneedi, PraveenOtto, JohnChládek, GustavOttoboni, MatteoChmelíková, EvaOtway, Nicolas M.Cho, JongkiOtzen, HenningCho, SohyunOuedraogo, Frederic B.Choct, MinganOuellet, VéroniqueChoi, ByungwoongOverall, KarenChoi, MincheolOverturf, KenChoi, Won HyungOviedo, EdgarChoi, Young MinOvissipour, RezaChong, RogerOvtchinnikov, IgorChou, Jen-YunOwen, Jeb P.Chow, Pizza Ka YeeOwens, FredChow, SeinenOzella, LauraChowdhury, Vishwajit S.Pacelli, CorradoChoy, EmilyPachas, NahuelChrenek, PeterPacheco, DavidChristensen, Bruce W.Pacheco, MárioChristensen, Janne WintherPadalino, BarbaraChristensen, RachaelPadhi, SoumeshChristie, StewartPadilla, DanielChu, MingxingPaes, Antonio CarlosChu, ThinhPaeshuyse, JanChud, TatianePaez, EzeChung, Wen-SungPagan, Joe D.Chwalibóg, AndréPageat, PatrickCiccarelli, MichelaPahm, SarahCiccarese, SalvatricePaillot, RomainCieslak, AdamPakhomova, OlgaCieślak, JakubPalacios, VicenteCilia, GiovanniPalade, Laurentiu MihaiCiliberti, Maria GiovannaPalanisamy, SatheeshkumarCinq-Mars, DanyPalazzo, MarisaCiocan, CorinaPalencia, JorgeCiotola, FrancescaPalestrini, ClaraCircella, ElenaPalin, Marie-FranceCitek, JindrichPalinski, RachelCiulu, MarcoPallotta, Nicole R.Claire, Wathes D.Palma Sircili, MarceloClaridge, Andrew W.Palma-Vera, SergioClark, EmilyPalme, RupertClark, FayPalmerini, MariaClark, Judy MacArthurPaludo, Giane ReginaClark, SherriePalus, KatarzynaClark, StephaniePan, ChuanyingClarke, NancyPan, JinmingClavenzani, PaoloPan, YuanlongClegg, Simon R.Panasiewicz, GrzegorzClemente, LurdesPandyaswargo, Andante HadiCleveland, Beth M.Panee, JunClutton, EddiePaneru, BamCoals, PeterPang, DanielCocchia, NatasciaPantaziz, PanosCocco, RaffaellaPaoletti, BarbaraCodd, Jonathan R.Paolini, AlessandraColazo, Marcos G.Paolucci, MarinaColditz, IanPapadakis, IoannisColeman, RobertPapadopoulos, Elias G.Colgan, Donald J.Papadopoulos, GeorgiosColle, MichaelPapadopoulos, SerafeimCollins, CourtneyPapakosta, Malamati A.Collins, KatiePapatsiros, Vassilios G.Collins, Michael D.Papich, Mark G.Collins-Emerson, JuliePapini, RobertoCollisson, EllenPapović, TamaraColombini, StefaniaParambeth, Joseph CyrusColpoys, JessicaParamio, María T.Columbano, NicoloParanhos Da Costa, Mateus José RodriguesColumbus, Daniel A.Paraskeuas, Vasileios V.Colussi, SilviaParcells, MarkComino, PennyParchem, Ronald J.Comizzoli, PierreParés-Casanova, Pere M.Comtat, ClaudeParisi, FrancescaConan, AnneParisi, GiulianaConn, David BruceParissi, Zoi M.Connor, Erin E.Park, Hee BokConnor, MelaniePark, Jae-HyungContalbrigo, LauraParkes, RebeccaConte, GiuseppeParolini, MarcoContiero, BarbaraParrilla, InmaContina, AndreaParrino, VincenzoContreras, GabrielaPartyka, AgnieszkaContreras-Aguilar, María DoloresPaschino, PietroCooke, BrianPascual, JuanCoombs, TamsinPascual, MariamCooney, KathleenPascual-Rico, RobertoCooper, IanPascuzzo, RiccardoCoppo, MauricioPasdeloup, DavidCoppola, CristaPasicka, EdytaCoppola, FrancescaPasquariello, RolandoCorassa, AndersonPasquini, MarinaCorazzin, MircoPassamonti, FabrizioCorbee, Ronald JanPassantino, AnnamariaCorbera, Juan AlbertoPassantino, GiuseppeCorbière, FabienPassero, FelipeCorda, AndreaPassler, ThomasCordoni, GiadaPassos, MarisaCords, MarinaPastore, CarloCorino, CarloPastorelli, GraziaCormier, NathalyPastorino, PaoloCornale, PaoloPatarata, LuísCornejo Kelly, JavieraPaterson, Gavin K.Corner-Thomas, Rene A.Paterson, MandyCorrêa Magalhães Filho, Fernando JorgePathakoti, KavithaCorrea, MaríaPatil, AnandCorreddu, FabioPatkowska-Sokoła, BożenaCorreia Caeiro, CatiaPatra, Amlan KumarCorreia, MiguelPatterson-Kane, Emily G.Corripio-Miyar, YolandaPatton, Robert A.Cortese, LauraPatzke, NinaCortes-Rodriguez, NandadeviPaudyal, SushilCosentino, CarloPaul, AmanCosma, ChiaraPaula, TilleyCosseddu, Gian MarioPausch, HubertCosta, AngelaPauselli, MarianoCosta, DiogoPavani, Krishna ChaitanyaCosta, ErnanePawar, KamleshCosta, Lais Rosa R.Pawlak, AleksandraCosta, Luciana DaPawlak, PiotrCosta, MarcioPayan-Carreira, RitaCostas, BenjaminPayne, StevenCostea, Paul IgorPaz Pellat, FernandoCosti, ElenaPaz-Silva, AdolfoCostilla, RoyPazzola, MicheleCoulson, GraemePeachey, LauraCoulter, KendraPearson, AngelaCoulthard, EmmaPease, Brent S.Cove, MichaelPeaston, AnneCove, Michael V.Pech Cervantes, Andres AlfredoCowl, VeronicaPechova, AlenaCozzi, AlessandroPecka-Kiełb, EwaCozzi, BrunoPedersen, Ken SteenCozzi, CristinaPedersen, Per AmstrupCozzoli, FrancescoPeffers, Mandy J.Craig, JessicaPegolo, SaraCrandell, KathleenPei, Kurtis Jai-ChyiCrapo, CharlesPeiretti, Pier GiorgioCravana, CristinaPelagalli, AlessandraCrawley, Jennie A. H.Peli, AngeloCregier, SharonPenaranda, IreneCrenshaw, ThomasPenedo, CeciliaCrépeaux, GuillemettePeng, YahuiCrescimbene, LaraPeng, ZuogangCrespo, FranciscoPenna, Bruno De AraujoCrespo-Piazuelo, DanielPenrith, Mary-LouiseCrisi, Paolo EmidioPerazzi, AnnaCristarella, SantoPerego, RobertaCristescu, BogdanPereira, BrunoCroft, David B.Pereira, FilipeCroitor, RomanPereiro, PatriciaCronin, Katherine A.Perera, ErickCrosta, LorenzoPerez Ibarra, IreneCrovetto, Gianni MatteoPerez, HectorCrowley, DanielPérez, José FranciscoCrowley, JamesPérez, ValentínCrowley, SarahPérez-Álvarez, Jose AngelCrowther, MathewPérez-Enciso, MiguelCrumière, Antonin J. J.Pérez-González, JavierCrump, AndrewPérez-Méndez, José A.Crupi, RosaliaPerez-Pe, RosauraCuchillo-Hilario, MarioPérez-Sánchez, TaniaCuello, CristinaPeriago, Maria VictoriaCuervo, BelénPeric, TanjaCuevas-Ramos, GabrielPeritore, Alessio FilippoCui, HuanxianPerkins, ElizabethCukrowska, BoženaPerkins, SarahCullen, BrendanPero, Maria ElenaCullere, MarcoPéron, GuillaumeCummins, Leo J.Perondi, FrancescaCunilleras, Eduard JosePerrault, Justin R.Curto, ManuelPerricone, VeraCutrignelli, Monica IsabellaPerrucci, StefaniaCzaczkes, Tomer JosephPerry, Elizabeth E.Czauderna, MarianPerry, ErinCzech, AnnaPerry, GadCzeglédi, LeventePersicce, Giorgio AntonioCzerniawska-Piątkowska, EwaPersson, KirstenCzeszczewik, DorotaPersson, YlvaCzyż, KatarzynaPertoldi, CinoD’Alessandro, Maria EugeniaPesavento, PatriciaD’Amico, MarcelloPesti, Gene M.D’Amico, RamonaPeters, AndrewD’Aniello, BiagioPeters, VerenaD’Argenio, ValeriaPetersen, HeidiD’Cotta, HelenaPetersen, JessicaD’Souza, DarrylPeterson, CarlynDa Luz E Silva, SauloPeterson, Karianne LievaartDa Silva Carvalho, JoanaPeterson, MichaelDa Silva, Lorrayny GaloroPeterson, SamDa̧browski, MichałPetitte, JimDabrowski, RomanPetow, StefanieDadousis, ChristosPetr, KotlíkDahl-Pedersen, KirstinPetra, SchnitzerDai, RuitongPetrera, FrancescaDai, XiaoxiaPetrizzi, LucioDalgaard, Tina SørensenPettey, Lee AllenDall’Aglio, CeciliaPfeffer, PeterDalla Costa, ManuelaPfeiffer, ChristinaDalla Villa, PaoloPfeuti, GuillaumeDalle Zotte, AntonellaPhilibert, DanielleDalloul, RamiPhysick-Sheard, Peter W.Dalmau, AntoniPiantino Ferreira, Antonio JoséDaly, KristianPiantoni, PaolaDalzini, ElenaPiao, XiangshuDam Otten, NinaPiccioli Cappelli, FiorenzoDam, Chinh Thi MyPiccione, GiuseppeDamaziak, KrzysztofPiccirillo, AlessandraDamiano, SaraPiccolo, GiovanniDamiran, DaalkhaijavPickett, AndyDang, RuihuaPickova, JanaDaniels, JaretPiechna, JanuszDaniels, SimonPieczywek, Piotr M.Dankowiakowska, AgataPienaar, RonelDanks, Janine A.Pieramati, CamilloDannenberger, DirkPierard, MarcDario, ColombattoPierce, EllenDark, MichaelPierce, KarinaDaros, Ruan R.Piercy, RichardDarre, Michael J.Pierdon, MeghannDarwish, AmiraPierre, CozannetDashper, KatherinePierri, FrancescaDasmahapatra, AsokPiersanti, SilvanaDastjerdi, AkbarPiestrzyńska-Kajtoch, AgataDatteri, EdoardoPieszka, MagdalenaDavid, González-BarrioPieszka, MarekDavidson, IritPietrock, MichaelDavies, DavidPietruszka, ArkadiuszDavies, MichaelPietsch, ConstanzeDavies, SimonPilarczyk, AndrzejDavin, RogerPilkington, GeoffreyDavis, Adam J.Pilla, RachelDavis, ChelseaPiluzza, GiannellaDavis, Donald AllenPinart, ElisabethDavis, JenniferPinheiro, VictorDavis, Mark A.Pinna, CarloDavis, Michael E.Pinotti, LucianoDavis, StevePintus, ElianaDavis, William C.Piórkowska, KatarzynaDavoudi, MasoumePiotti, PatriziaDawkins, Marian StampPiprek, Rafal P.Dawood, Mahmoud A.O.Piquer, OlgaDawson, LaurenPires, EuclidesDe Abreu, Luiz CarlosPires, OsmindoDe Almeida Bicudo, Álvaro JoséPirhonen, JuhaniDe Almeida, André M.Piscopo, MarinaDe Araújo Oliveira, Chiara AlbanoPistollato, FrancescaDe Araújo, José Pedro PintoPitesky, Maurice E.De Biase, DavidePiva, AndreaDe Boni, Annalisa De BoniPiwczyński, DariuszDe Briyne, NancyPiwetz, SarahDe Camargo, Gregório Miguel FerreiraPizarro, ManuelDe Carvalho, Gleidson Giordano PintoPla-Aragones, LuisDe Castro, Ana Catarina VieiraPlachá, IvetaDe Castro, Ignacio PerezPlain, KarrenDe Clercq, DominiquePlanas Oliver, MiguelDe Felice, ElenaPlascencia, AlejandroDe Figueirêdo Freitas, Vicente JoséPłoneczka-Janeczko, KatarzynaDe Girolamo, PaoloPlowman, JeffDe Jesús Ruíz-López, FelipePocrnic, IvanDe La Fe, ChristianPoeggeler, Burkhard H.De La Fuente Oliver, GabrielPoglayen, GiovanniDe La Fuente Vázquez, JesúsPogorzelska-Nowicka, EwelinaDe La Fuente, GabrielPoh, Karen C.De Lagarde, MaudPokorny, BoštjanDe Lima, Valéria Marçal FelixPolak, GrażynaDe Los Reyes, MónicaPolak-Śliwińska, MagdalenaDe Majo, MassimoPolasik, DanielDe Mori, BarbaraPoley, Lucy G.De Oliveira, Marcos RobertoPolidori, PaoloDe Palo, PasqualePolizopoulou, ZoeDe Paula, Eduardo MarosteganPollard, DanicaDe Queiroz Prado, RafaelPollera, ClaudiaDe Rensis, FabioPollitt, ChrisDe Rosa, GiuseppePollo, SimoneDe Silva, SherminPombo, AnaDe Souza Gil, EricPomerantz, OriDe Souza, Danival JoséPomeroy, Kimball O.De Vere, AmberPongrácz, PéterDear, Jonathan DavidPonnampalam, EricDebelica-Lee, AnicaPonte, GiovannaDebertolis, PaoloPonzetti, MarcoDeblanc, CélinePonzoni, RaulDeblitz, ClausPoock, ScottDębski, BogdanPopescu, SilvanaDed, LukasPorr, SheaDeflorio, MichelePortas, TimDegani, GadPosautz, AnnikaDegl’Innocenti, SaraPotes, Maria EduardaDego, Oudessa KerroPotočnik, KlemenDeGregorio, BrettPouil, SimonDegroote, JeroenPourlis, ArisDehoux, Jean-PaulPoutahidis, TheofilosDel Buono, DanielePovilitis, AnthonyDel Pino, Francisco Augusto BurkertPowell, LaurenDel Valle, Tiago AntonioPowell, MattDelgado Bermejo, Juan VicentePowers, Amanda K.Delgado, Juan VicentePrabhu, LakshmiDelgado-Bermúdez, AriadnaPrado, Ivanor Nunes DoDelia, LacastaPrado, María RodríguezDell, AndyPrado-Oviedo, Natalia A.Dell, ColleenPramod, SreepriyaDell’Aqua Junior, José AntonioPrange, TimoDell’Era, PatriziaPravettoni, DavideDella Casa, GiacintoPrearo, MarinoDellamaria, DeboraPreez, Surita DuDellinger, MyannaPresto Åkerfeldt, MagdalenaDelon, NicolasPreziuso, SilviaDelwart, EricPriante, GiovannaDeMarais, AlycePřibylová, LucieDeMello, MargoPrice, ChristopherDemyda, SebastianPrieto, MiguelDemyda-Peyrás, SebastiánPringle, John K.Denis, ChristophePrins, Jan-BasDenk, DanielaPrischi, FilippoDenkova-Kostova, Rositsa StefanovaPriyadarshana, ChathuraDennis, Rachel L.Prochazka, RadekDenzin, NicolaiProroga, Yolande Thérèse RoseDeputte, BertrandProsser, Colin G.Derks, Martijn F. L.Proszkowiec-Weglarz, MonikaDervishi, EldaProtopopova, AlexandraDesantis, SalvatoreProudfoot, KatyDeschamps, Jack-YvesProwle, MalcolmDescovich, KrisPrzysławski, JuliuszDetilleux, JohannePsomas, AkisDetry, CleiaPtacek, MartinDettori, Maria LuisaPucéat, MichelDevant, MariaPuggioni, AntonellaDevriendt, NausikaaPugliese, CarolinaDhakal, SantoshPugliese, MichelaDhanasiri, AnushaPulido, Rubén GuillermoDi Bella, SantinaPulina, GiuseppeDi Bello, AntonioPullen, NicholasDi Bene, ClaudiaPuppel, KamilaDi Fiore, Maria MaddalenaPurdy, Phil H.Di Francesco, AntoniettaPurwin, CezaryDi Giancamillo, MauroPüschel, ThomasDi Girolamo, NicolaPutman, RoryDi Grigoli, AntoninoPuurunen, JenniDi Iorio, MichelePuvača, NikolaDi Martino, GuidoPuzio, IwonaDi Pasquale, JorgelinaQi, GuanghaiDi Profio, FedericaQiao, ShiyanDi Stasio, LilianaQin, YiminDi Trana, AdrianaQiu, Hua-JiDiana, AlessiaQu, KaixingDiane Valeriano, ValerieQu, MingziDiao, QiyuQuandt, JaneDias, Maria Isabel RibeiroQuaranta, AngeloDias-Pereira, PatriciaQuartuccio, MarcoDiaz De Cerio, OihaneQudsieh, RashaDíaz Gaona, CiprianoQueiroga, FelisbinaDíaz, MarioQueiros, JoãoDickman, ChrisQuéré, PascaleDicksved, JohanQuilez, JoaquinDiCostanzo, AlfredoQuinn, NiamhDiéguez, Francisco JavierQUINSAC, AlainDierenfeld, Ellen S.Quintana, Álvaro RafaelDierick, NoëlQuinteiro-Filho, Wanderley MorenoDiez, RaquelRabalski, LukaszDiggles, BenjaminRadaelli, GiuseppeDillon, JasmineRadcliffe, Rolfe M.Dimauro, CorradoRadko, LidiaDimitrov, KirilRadkowska, IwonaDing, LumingRadzevičienė, AurelijaDing, YuRadzik-Rant, AureliaDini, PouyaRaedts, Peter J.M.Dinis, MárioRafales, Cristina BonastreDistl, OttmarRaffan, EleanorDistler, ClaudiaRaffrenato, EmilianoDiverio, SilvanaRaftery, MartinDixon, LauraRagan, IzabelaDjokovic, RadojicaRagkos, AthanasiosDo, Duy NgocRagni, MarcoDobos, RobinRahimnejad, SamadDobrowolski, PiotrRahman, Inayat UrDodds, W. JeanRaina, SatishDohme-Meier, FriggaRainho, AnaDoi, HideyukiRaj, EldonDoligalska, MariaRaj, MohanDolinská, MichaelaRajko-Nenow, PaulinaDolka, IzabellaRajput, Mrigendra K.S.Domínguez Rebolledo, Álvaro EfrénRajput, SandeepDomínguez, JesúsRak, AgniezkaDominguez, Juan CarlosRakov, AlexeyDomínguez-Bernal, GustavoRamachandran, AnupDomínguez-Pérez, DanyRamalho-Santos, JoãoDomínguez-Vías, GermánRamchandra, RohitDominik, SonjaRamírez Rivero, Gustavo A.Domino, MalgorzataRamirez, BrettDonadelli, Renan A.Ramirez, ChristinaDong, LifengRamírez-Avilés, LuisDonnay, IsabelleRamirez-Bribiesca, J. EfrenDonnini, SandraRamírez-Mella, MónicaDorado, JesúsRamiro Rodriguez, ManuelDoran, TimothyRamón, ManuelDorey, NicoleRamos, Eric A.Dörgeloh, WernerRamos, FernandoDorin, CraigRampacci, ElisaDorman, David C.Rand, JacquieDorny, PierreRandazzo, BasilioDors, ArkadiuszRandhawa, ImtiazDorsch, RoswithaRandle, HayleyDorszewski, PiotrRando, AndreaDos Santos Sousa, Carlos AugustoRapetti, LucaDos Santos, Fábio A.AbadeRasero, RobertoDos Santos, Regiane RodriguesRaskovic, BozidarDos Santos, Tatiana CarlescoRasmussen, Martin KrøyerDotsev, ArsenRasmussen, Morten DamDoughty, AmandaRasmussen, Sophie LundDouglas, CatherineRaspa, FedericaDouglass, RickRathgeber, BruceDowgier, GiuliaRatnayake, ChamindaDowling, SeanaRato, Luís Pedro FerreiraDowning, JeffreyRatto, MarceloDowns, KevinRauch, ElkeDoyle, EmmaRaulf, MonikaDozza, BarbaraRavera, IvanDrażbo, Aleksandra AlicjaRavetto Enri, SimoneDrewnowska, OlgaRavindran, VelmuruguDriessen, BertRaw, ZoeDrillich, MarcRawlings, John MerrittDroscha, CaseyRawski, MateuszDrozdov, AnatoliyRay, ParthaDróżdż, TomaszRaynor, EdwardDryden, GordonReantaso, MelbaDu, FuliangRebordinos González, LaureanaDu, HuahuaRecio-Menéndez, ManuelDuan, YehuiRecuero, SandraDuarte, Marcio S.Reddy, Chandan G.Ducatelle, RichardReed, De MetrisDuchemin, SandrineReese, Laura A.Duckett, SusanReeve, NigelDuda, NiklasRegmi, PrafullaDuff, Glenn C.Regolin, LuciaDuffy, DavidReichel, Michael P.Dufrasne, IsabelleReicher, VivienDuggan, VivienneReid, ScottDuncan, NeilReifinger, MartinDunisławska, AleksandraReimert, HennyDunner, SusanaReina, RamsesDunshea, FrankReiner, GeraldDüpjan, SandraReis, Joana Da CostaDuplessis, MélissaReis, Mariza G.Dupré, Gilles P.Reis, Ricardo AndradeDuran, CarolinaReiss, MichaelDurán, Manuel NovalesRekaya, RomdhaneDuran, Sue HudsonRelling, Alejandro E.Durham, Amy C.Rempel, LeaDurham, AndyRen, LinzhuDurland, EvanRendo Fornet, FernandoDurmic, ZoeyResch, UlrikeDurrant, Barbara S.Ressel, LorenzoDurward-Akhurst, Sian A.Rettedal, ElizabethDuszewska, AnnaReverter, MiriDutta, RahulReverter, MiriamDuvnjak, MarijaReyer, HenryDybus, AndrzejReynolds, JimDyer, JimRezamand, PedramDyson, SueRezar, VidaDzidic, AlenRiad, BouzidDziekońska, AnnaRibani, AnisaDżugan, MałgorzataRibeiro, Maria NormaEastwood, Justin R.Ribitsch, IrisEbani, Valentina VirginiaRichardson, IanEbensperger, GermánRichardson, Michael K.Ebertz, PeterRichomme, CélineEckmeier, DennisRichter, KatharinaEddison, JohnRickards, KarenEdes, Ashley N.Rico, CarlosEdinboro, Charlotte H.Rico, DanielEdokpayi, Joshua NosaRico, EduardoEdwards, Adrianne N.Riek, AlexanderEdwards, Gaylen L.Riesco, Marta F.Edwards, Katie L.Rilcheva, Violeta S.Edwards, Lauren E.Riley, ChristopherEdwards, PhoebeRiley, David GregEdwards, SandraRiley, Lisa M.Edwards-Callaway, LilyRiley, SophieEgea, Pascal F.Rimoldi, SimonaEggel, MatthiasRinaldi, LauraEggertsen, GostaRinnovati, RiccardoEgyed, LászlóRipoll, GuillermoEhmcke, JensRitler, DominicEik, Lars OlavRitskes-Hoitinga, MerelEinspanier, RalfRiva, AlessandraEiras, JorgeRiva, FedericaEisenberg, SusanneRivera Del Álamo, Maria MontserratEkker, MarcRivera, DanielEl Hage, CharlesRivero Viera, Myriam JordanaElbers, Armin R.W.Rivero, José Luis LópezEleyadeth-Meethal, MuhammedRiveros, José LuisElguezabal, NataliaRizvi, Syed Husain MustafaElias, MiguelRizzi, ChiaraElizur, AbigailRoana, JaniraElleder, DanielRobbins, LindseyEllerbrock, RobynRoberto Da Costa, Rosário PlócidoEllingsen-Dalskau, KristianRoberto Da Silva, EdsonEllis, Andrea DorotheaRoberts, DaleEllis, WilliamRoberts, Juliet R.Elmahallawy, EhabRobertson, AndyElmi, AlbertoRobertson, SusanElokil, AbdelmotalebRobic, AnnieElolimy, AhmedRobins, AndrewEl-Sheikh Ali, HossamRobinson, CharlotteElsholz, SabrinaRobinson, Kelly J.Elsohaby, IbrahimRobinson, KelsyElvang Jensen, HenrikRoca-González, José LuisElwan, HamadaRocchigiani, GuidoElwood, Robert W.Rocha, Luiene M.Elzanowski, AndrzejRoche, StevenEmerenciano, Maurício Gustavo CoelhoRock, Melanie J.Encinar, Jorge RuizRodenburg, JackEncinas, TeresaRodero Serrano, EvangelinaEnders-Slegers, Marie JoséRodger, JohnEngelken, TerryRodrigo Mocholi, DiegoEnger, Benjamin D.Rodrigo-Comino, JesusEngvall, Eva OlssonRodrigues Da Costa, MariaEric, PaquetRodríguez De Cara, María ÁngelesErickson, PeterRodriguez Ferrere, MarceloErikson, DavidRodriguez, KerriErlwanger, Kennedy H.Rodríguez, MartínErram, DineshRodríguez, Virginia GaminoEspinosa, CharmaineRodríguez-Estévez, VicenteEspinosa, VíctorRoelfsema, EllenEssen, Erica VonRogers, Chris W.Essler, Jennifer L.Rogers, JeffreyEssner, AnnRogiewicz, AnnaEsteban, Ma. AngelesRogovskyy, ArtemEsteve-Garcia, EnricRöhe, IlenEstrada, Jose L.Rois, DiegoEstrada-Angulo, AlfredoRolbiecki, LeszekEusebi, Paulina GarcíaRolinec, MichalEvans, NeilRollin, Bernard E.Evans, RhysRomagnoli, StefanoEven, NaïlaRomano, AndreaEwald, VincentiusRomano, NicholasFabbri, GiorgiaRomans, Emma Fàbrega IFabbri, Maria ChiaraRomar, RaquelFabrizio, EnricoRoncarati, AlessandraFabrizio, MauroRondina, DavideFaciola, AntonioRönnegård, LarsFadok, ValerieRooney, NicolaFahey, George C.Roos, Anna MarieFajt, Virginia R.Roos, JulietteFaleiros, RafaelRoosenburg, WillemFan, Ming Z.Ropka-Molik, KatarzynaFan, ZhiyongRoques, JonathanFanelli, AngelaRørvang, Maria VilainFaragó, TamásRosado, BelénFarmer-Dougan, ValeriRosales, RubenFarnir, FrédéricRosati, LuigiFarnworth, Mark J.Rose, PaulFarouk, Mohammed HamdyRosenberg, MosheFarzan, VahabRoss, JamesFassani, Édison JoséRoss, SteveFassina, AmbrogioRos-Santaella, José LuisFattorini, NiccolòRossetti, Carlos A.Faucitano, LuigiRossi, LucaFaulkes, ZenRossi, RaffaellaFaustini, MassimoRosso, Alessia DiFaustino, AnaRossow, HeidiFawcett, AnneRota, AdaFayad, ZahiRoth, LinaFazio, EsterinaRotherham, Ian D.Fazio, FrancescoRotllant, Guiomar E.Feddern, VivianRouger, AmélieFei, Andrew Chang-YoungRoura, XavierFeito, JavierRoussel, AllenFelix, AnandaRovai, MaristelaFélix, LuisRowan, AndrewFeliziani, FrancescoRowe, AnthonyFeng, WanjuanRowe, MelissahFenner, KateRoy, CyrilFenton, BrockRozanski, Elizabeth A.Feregrino-Pérez, Ana A.Rubenstein, Dustin R.Ferenc, PajorRubessa, MarcelloFerguson, James D.Rubio Lozano, Maria SaludFerlazzo, AdrianaRuczizka, UrsulaFerlizza, EneaRudeck, JulianeFernandez, EduardoRudi, KnutFernandez, IgnacioRueca, FabrizioFernández, JesúsRuelle, ElodieFernández, MiguelRuet, AliceFernández-Alacid, LauraRufener, ChristinaFernández-Bellon, HugoRugani, RosaFernández-Robledo, José AntonioRumbos, Christos I.Fernando Ivan Flores, PerezRussell, James R.Fernando, DeepaniRutberg, Allen T.Ferneborg, SabineRutgen, BarbaraFerra, BartłomiejRuviaro, Clandio FavariniFerramosca, AlessandraRužauskas, ModestasFerrante, ValentinaRyan, EoinFerraz, MarciaRybczyk, AgnieszkaFerraz, PatriciaRybska, MartaFerreira Dias, GracaRychlik, IvanFerreira, Ana CristinaRyczek, NataliaFerreira, SusanaRyman, Valerie E.Ferreira, Vitor Hugo BessaRyndock, EricFerreras, Javier CañónRypuła, KrzysztofFerrer-Pérez, HugoRząsa, AnnaFerro, SilviaRzepkowska, MałgorzataFetherman, Eric R.Saadeldin, IslamFeuerbacher, EricaSaastamoinen, MarkkuFeyera, TakeleSabbioni, AlbertoFidalgo, MiguelSabia, EmilioFigueroa Millán, Julio V.Sachin, SharmaFigueroa, JaimeSacks, MurielFilipello, VirginiaSady, MarekFinch, DomhnallSaelao, PerotFine, Aubrey H.Saenz De Juano Ribes, MaraFinelli, CarmineSáenz, LeonardoFinka, LaurenSáez Casado, María IsabelFinke, MarkSafranski, TimothyFink-Gremmels, JohannaSaha, SudebFiore, EnricoSahagun, Ana M.Fioretti, AlessandroSahlmann, ChristianFischer, TedSahni, Sanjeev K.Fischer-Tenhagen, CarolaSaidenberg, AndréFiset, SylvainSaitoh, KenjiFisher, DanielSakami, TomokoFitzpatrick, JulieSakayori, NobuyukiFitzsimmons, SuzanneSakomura, Nilva KazueFleming, Patricia A.Sakowski, TomaszFlint, SteveSakudo, AkikazuFlis, MarianSalak-Johnson, Janeen L.Flisikowski, KrzysztofSalama, Ahmed A.K.Florek, MariuszSalas, MarinaFlythe, MichaelSalavati, MazdakFocken, UlfertSalazar-Villanea, SergioFodor, LászlóSaldaña-Vázquez, Romeo A.Fondevila, ManuelSalem, Abdelfattah Z.M.Fonseca Silva, FabyanoSaleri, RobertaFonseca, PabloSales Baptista, ElviraFontbonne, AlainSalgado, Luis MiguelForbes, AndrewSalinas, HomeroFord, LaurenSalles, Márcia S. V.Ford, MarkSaloña-Bordas, Marta I.Fordyce, GeoffrySalvarina, IoannaForeman, Jonathan H.Salzano, AngelaForghani, FereidounSamaras, AthanasiosForkman, BjörnSamardžija, MarkoFormaggioni, PaoloSamet, LaurenForman, DanielSamiec, MarcinFormoso-Rafferty, NoraSamková, EvaForonda, PilarSamolińska, WiolettaForrest, RachelSamoy, YvesForte, ClaudioSampaio, José PauloFortina, RiccardoSamuel, RyanFoskolos, AndreasSan Juan Serrano, FuencislaFoster, DerekSánchez Quinteiro, PabloFournier, AngelaSánchez Saavedra, María Del PilarFowler, Ashley L.Sanchez, CeciliaFoyle, LeoSanchez, Marie-PierreFraile, LorenzoSánchez, Marta GarcíaFrajblat, MarcelSanchez, Nicole C. BurdickFraley, Gregory S.Sánchez-Ajofrín, IreneFranch, JordiSánchez-Escalante, ArmidaFranciosi, ElenaSánchez-Guerrero, María JoséFranciosini, MariaSánchez-Nuño, SergioFrancisco, Alexandra E.Sanchis, JaimeFranco, Carlos M.Sander, SaaraFranco, MarciaSandmeier, Fran C.Franco, Nuno HenriqueSandøe, PeterFranco-Martinez, LorenaSandor, ZsuzsannaFranczak, AnitaSandoval-Castro, CarlosFrank, KrisztiánSaneyasu, TakaokiFrank, LaurenceSantana, AdinaFrankel, TylerSantana, Mário L.Franklin, DonSantaniello, AntonioFrant, MaciejSantiago-Moreno, JuliánFranzoi, MarcoSantini, AntonelloFraser, LeylandSantonicola, SerenaFraslin, ClemenceSantoro, DomenicoFratini, FilippoSantos, Ana SofiaFrazzon, AnaSantos, Andreia A.F.Frean, JohnSantos, Carla S.Freeman, Carrie PackwoodSantos, Geraldo TadeuFreeman, Elizabeth WatsonSantos, José MariaFregonesi, José AntonioSantos, LuanFrei, BarbaraSantos, Natalia R. DosFreire, RafaelSantos, Virgínia Alice Cruz DosFreitas Junior, José EslerSantos-Gomes, GabrielaFreitas, Patrícia D.Santos-Jimenez, ZurisadayFreitas, RenataSantos-Silva, JoséFreking, Bradley A.Sanz-Paris, AlejandroFrey, Jennifer K.Sapountzis, PanagiotisFrias, JoaoSapudom, JiranuwatFrias, RafaelSarabia, JaimeFricke, PaulSaraiva, João L.Frieden, MaudSarauer, JessicaFriend, TedSarmento, Jose Lindenberg RochaFritts, MarkSarmiento-Franco, LuisFröhlich, MarlenSaro, CristinaFroment, PascalSarti, Francesca MariaFronte, BaldassareSartori, CristinaFrosini, SianSasal, PierreFrossard, Jean-PierreSato, HiroshiFry, Lindsay M.Sato, ShingoFrye, ChristopherSatou, TadaakiFrylinck, LorindaSatta, YokoFrynta, DanielSattler, WolfgangFthenakis, GeorgeSattley, W. MatthewFu, BeideSatué, KatiuskaFugazza, ClaudiaSauerwein, HelgaFujimori, KoSaurav, KumarFukami, KimioSavelkoul, Huub F.J.Fuller, Frederick J.Savell, JeffFuller, GraceSavic, VladimirFunahashi, HiroakiSavoca, SerenaFurre, SiriSavvas, IoannisFurstenau, Tara N.Sawicka-Zugaj, WiolettaFuruita, HirofumiSawyer, Jason E.Fusaro, IsaSawyer, Jason T.Fusco, RobertaSayyari, AminFusellier, MarionScala, AntonioFusi, FrancescaScandurra, AnnaFusté-Forné, FrancescScanes, Colin G.Fyfe, JohnScerra, ManuelGäbel, GottholdSchaefer, Allan L.Gabler, ChristophSchäfer, Sarah K.Gácsi, MártaSchäfer-Somi, SabineGadea, JoaquinSchalles, MattGaffuri, AlessandraSchedle, KarlGagaoua, MohammedScheffler, JasonGai, FrancescoScheiner, RicardaGajewski, ZdzislawScherk, MargieGalarce, NicolásSchiavone, AchilleGalassi, GianlucaSchild, Sarah-Lina AagaardGalatos, Apostolos D.Schiliger, LionelGałęcki, RemigiuszSchinckel, Allan P.Gálik, BranislavSchindler, MariaGalisteo Junior, Andres J.Schlecht, EvaGalkina, Svetlana A.Schmid, MarkusGallardo, Carolina MunozSchmitt, ArminGallardo, María A.Schmitt, OceaneGallego Albiach, VictorSchmoelzl, SabineGallegos-Funes, FranciscoSchmolz, ErikGallina, SoniaSchmucker, SonjaGallo, CarmenSchneider, JiříGallo, LuigiSchoeman, JohanGambini, MatteoSchofield, GailGandolfi, FulvioSchoiswohl, JuliaGandra, JeffersonSchokker, DirkjanGantner, MagdalenaScholz, ArminGao, ChunqiSchook, Mandi WilderGao, SongSchoster, AngelikaGao, YouheSchott, Harold C.Garami, AndrasSchrader, LarsGarbossa, Cesar Augusto PospissilSchrama, DeniseGarces Narro, CarlosSchrey, AaronGarcía Alvarado, José SantosSchroeder, Jennifer R.Garcia Alvarez, OlgaSchubbert, AntjeGarcia Ispierto, IrinaSchulte, Bruce AlexanderGarcía Mesa, SergioSchulze-Tanzil, GundulaGarcia Pardo, Maria De La LuzSchusser, Gerald FritzGarcía Rebollar, PilarSchütz, Luís FernandoGarcia, AntonioSchutz, MichaelGarcia, ArleneSchwab, NielsGarcía, JavierSchwarz, DanielGarcia, Lyda G.Schwarz, TomaszGarcía, María-LuzSchwarzenberger, FranzGarcia, MatthewSchwarzer, AngelaGarcia, MichelleSchweizer, MatthiasGarcía-Álvarez, OlgaScillitani, GiovanniGarcía-Belenguer, SylviaScior, ThomasGarcía-Casco, JuanScobie, DavidGarcia-Estañ, Luis PerezScocco, PaolaGarcía-García, Rosa MaríaScofield, RoseGarcía-Gasca, Silvia AlejandraScoley, GillianGarcía-López, RodrigoScollo, AnnalisaGarcia-Mazcorro, JoseScopa, ChiaraGarcia-Perez, Ana LuisaScorpio, DianaGarcía-R, Juan CarlosScott, JacquelineGarcía-Rebollar, PalomaSeal, BruceGarcía-Rodríguez, AserSebastian, CanovasGarcía‑Vázquez, Francisco A.Secci, GiuliaGard, Julie A.Sechi, Leonardo A.Garde, José JuliánSeco-Rovira, VicenteGardón, Juan CarlosSeeth, Martin ThoGarippa, GiovanniSegat, Hecson JesserGarmyn, Andrea J.Segura Plaza, José FranciscoGarner, JosieSehgal, InderGarnham, LauraSeidavi, AlirezaGarnsworthy, PhilSekiguchi, SatoshiGarofalo, CristianaSekino, MasashiGarofalo, LuisaSell-Kubiak, EwaGarrels, WiebkeSeltmann, Martin W.Garrido-Fernández, AntonioSelvaggi, MariaGarstang, MichaelSembratowicz, IwonaGasco, LauraSemlali, AbdelhabibGąsior, RobertSenczuk, GabrieleGaspa, GiustinoSénèque, EmilieGaspar, AugustaSeniczak, AnnaGáspár, RóbertSenigaglia, ValeriaGasparrini, BiancaSeoane-Collazo, PatriciaGatel, LaureSepe, LuciaGates, RichardSepulveda, NéstorGatto, FrancescaSeradj, Ahmad RezaGautam, MukeshSerefko, AnnaGautron, JoëlSerieys, Laurel E.K.Gawalek, JolantaSerna, EvaGawlik, Kinga IzabelaSeroussi, EyalGaworski, MarekSerrano, EmmanuelGazzano, AngeloSerrano-Montes, José LuisGazzola, AndreaSerrano-Perez, BeatrizGazzonis, Alessia LiberaSerrapica, FrancescoGebhardt-Henrich, SabineServais, VéroniqueGehman, Alyssa Lois M.Sessions-Bresnahan, Dawn R.Geist, JuergenSeth-Smith, HelenaGekenidis, Maria-TheresiaSevi, AgostinoGelsleichter, James J.Sewint, NancyGenchi, ClaudioSeyfang, JemmaGentz, MariaSgorbini, MicaelaGeraldo, AnaShaffer, GaryGerencsér, ZsoltShah, VrutantGermana, AntoninoShaham, YavinGerritzen, MarienShakeri, MajidGertz, MarvinShamieh, SaidGesek, MichałShamshiri, Redmond R.Gethöffer, FriederikeShamsi, ShokoofehGetty, KellyShanmugam, MuruganandanGhanem, KahinaSharafeldin, TamerGhassemi Nejad, JalilSharara, MahmoudGhidini, MicheleSharma, ArvindGhosh, AjitSharp, ClaireGhosh, SharmilaSharpe, Lynda L.Ghuman, Sarvpreet SinghShaw, AndrewGiacomo, GnudiShearer, Jan KeithGiadinis, Nektarios D.Sheehan, DiarmuidGialletti, RodolfoSheen, Shyn-ShinGiammarco, MelaniaShehadeh, KaskousGiangaspero, MassimoShelomi, MatanGiannenas, IliasShen, JunshiGiannetto, AlessiaShen, LimingGiannetto, ClaudiaShepherd, MeganGiarratana, FilippoShepley, EliseGibson, JohannaSheriff, MichaelGifford, RobertShi, FangxiongGil, Maria AntoniaShi, HengboGilbert, Robert O.Shi, QiongGiller, KatrinShi, ZhendanGines Ruiz, RafaelShi, ZhengxiangGioia, GloriaShih, Hao YuGiovambattista, GuillermoShike, Daniel W.Giráldez, Francisco JavierShim, Kwan SeobGiráldez-Diaz, InmaculadaShimizu, MikiGiri, BanabihariShiraishi, Jun-ichiGiri, Sib SankarShiu, YuGirolami, FlaviaShivley, Chelsey B.Girondot, MarcShivley, JakeGisbert, EnricShree, SonalGisondi, SilviaShuai, SurongGispert, MarinaShumskaya, Alena EuhenaunaGiudice, ElisabettaShusuke, SatoGiuliotti, LorellaShuttleworth, CraigGiuseppe, ArcangeliSidiropoulou, ErasmiaGlade, Michael J.Siegel, Paul B.Gladue, DouglasSiemieniuch, Marta J.Glassey, SteveSiettou, ChristinaGlatz, PhilSignal, TanyaGlen, Alistair S.Sih, AndrewGlendinning, LauraSikora, AnnaGlenk, Lisa MariaSilacci, PaoloGlennon, MichaleSilas Fernandes, EtoGlikman, JennySillero, NeftalíGłogowski, RobertSilva, Alexandre RGmel, Annik ImogenSilva, D.D.Gobikrushanth, MohanathasSilva, EdianeGoddard, EllenSilva, Josineudson Augusto Ii VasconcelosGoddard, PeteSilva, KarineGodfrey, Robert W.Silva, LuizGodia, Marta.Silva, Severiano R.Godwin, KrisSilva-Rodríguez, Eduardo A.Godyń, DorotaSilvestrelli, MaurizioGoerlich-Jansson, VivianSilvi, StefaniaGoetsch, Arthur LouisSilvia, BonardiGoiri, IdoiaSimintiras, ConstantineGołaś, IwonaSimitzis, PanagiotisGolbeck, LennartSimões, João Carlos CaetanoGoleman, MałgorzataSimon, AnetteGolomazou, EleniSinclair, MichelleGoluch, ZuzannaSinger, EllenGómez Carpio, Mayra M.Singer, RandallGomez, ErnestoSinger, Steven M.Gomez, Forrest M.Singh, BhupinderGómez, MarceloSingh, ParamGomez-Casado, EduardoSingh, YazavinderGomez-Piñero, EnriqueSinha, AmitGomis, JesúsSiniscalchi, MarcelloGonçalves, PedroSinski, JenniferGonçalves, SóniaSion, GuyGonella-Diaza, Ángela MaríaSips, PatrickGong, JinyanSiqueira, FrancieleGonzalez Montana, Jose RamiroSiracusa, CarloGonzalez, GabrielSirri, FedericoGonzalez, John MichaelSitkowska, BeataGonzalez, ManuelSivalingam, PeriyasamyGonzález-Barrio, DavidSiwek, MariaGonzalez-Bulnes, AntonioSjölund, MarieGonzalez-Casado, AntonioSkarzynski, Dariusz JanGonzález-Doncel, MiguelSkowron, KrzysztofGonzález-Fernández, LauroSkowroński, Mariusz TomaszGonzález-Martín, Juan V.Skřivan, MilošGonzález-Martínez, ÁngelaSkřivanová Eva, EvaGonzález-Montaña, José RamiroSkrlep, MartinGonzález-Ramírez, Mónica TeresaSkrzyszowska, MariaGonzález-Redondo, PedroSkylas, DanielGonzález-Ríos, HumbertoSlade, Joel W.G.Gonzalez-Vicente, AgustinSladky, KurtGonzalo, CarlosŚlaska, BrygidaGood, Deborah J.Slater, MargaretGoodband, Robert D.Slotta-Bachmayr, LeopoldGoodla, LavanyaSłowińska, MariolaGoodrich, ErinSloyer, KristinGoossens, KarenSłupecka-Ziemilska, MonikaGopegui, Rafael R.Smaldone, GiorgioGordon, JessicaSmall, AlisonGordon, StuartSmall, Brian C.Gorgoglione, BartolomeoSmarsh, DanielleGörlich, DennisŚmiałek, MarcinGórski, KrzysztofSmith, AdamGort, GerritSmith, DonaldGorzelak, PrzemysławSmith, GrahamGossman, ToniSmith, IanGoto, TatsuhikoSmith, KenGöttert, ThomasSmith, Nicholas C.Gottwald, EricSmith, Todd G.Gougoulis, Dimitris A.Smith, W. BrandonGould, Phillip S.Smith, ZacharyGouveia, KellySmołucha, GrzegorzGoyache, FélixSneddon, JenniferGoździewska-Harłajczuk, KarolinaSnowdon, CharlesGraaf, Simon DeSobek, ZbigniewGrabner, Daniel S.Sobiech, PrzemysławGraham, LauraSobolewski, JarosławGraham, Melanie L.Socha, MagdalenaGrahofer, AlexanderSoglia, DomingaGralak, Mikołaj AntoniSokolowski, MichelGrande, RaffaeleSolà-Oriol, DavidGrandin, TempleSolcan, CarmenGranica, SebastianSole-Guitart, AlbertGranquist, Erik GeorgSoler, MartaGrau, CarlosSolovyev, NikolayGray, CarolSommer, SylwesterGreef, Jörg MichaelSommerfeld, VeraGreen, ClaySondgeroth, KerryGreenwood, Alex D.Song, TongxingGreguła-Kania, MonikaSong, ZhigangGreiman, StephenSongsasen, NucharinGreiner, LauraSooryanarain, HariniGrela, Eugeniusz RyszardSørensen, Sune RiisGrieco, ValeriaSoriano, Ana IsabelGriffiths, CharlesSoriano-Úbeda, CristinaGriffiths, Stephen R.Sossidou, EvangeliaGriffitts, CarolineSotira, StefaniaGrigg, Emma K.Soto, EstebanGrilli, EsterSoto-Rodriguez, Sonia A.Grilli, GuidoSouillard, RozennGrimard, BenedicteSoulsbury, Carl D.Grimm, HerwigSoumeh, Elham AssadiGrippi, FrancescaSoyeurt, HélèneGrispoldi, LucaSpada, EvaGrodzik, MartaSpadari, AlessandroGrolli, StefanoSpadola, FilippoGröndahl, GittanSparks, ConradGruen, Margaret E.Spence, KelseyGrugan, ShannonSpengeler-Frischknecht, MirjamGrün, Nicole SommerSpengler Neff, AnetGrund, ChristianSpergser, JoachimGruszczyńska, JoannaSperoni, MarisannaGruszecki, TomaszSpielman, DerekGryz, JakubSpiers, DonGrzegorzewski, WaldemarSpindler, BirgitGrzybek, MaciejSpinella, GiuseppeGu, XianhongSpinu, MarinaGuan, WutaiSponenberg, PhillipGuan, YongjuanSpringer, SvenjaGuaratini Ibelli, Adriana MerciaSquire, SylviaGuardiola, Francisco A.Squires, E. JamesGuberti, VittorioStaaks, GeorgGuelfi, GabriellaStachowicz, AnetaGuenther, Katja M.Stachurska, AnnaGuérios, Simone DomitStadig, Lisanne M.Guerreiro, OlindaStadnicka, KatarzynaGuest, DebbieStadtlander, TimoGugliandolo, EnricoStalker, LeanneGuglielmini, CarloStancampiano, LauraGugołek, AndrzejStanek, MagdalenaGuimaraes, Simone E.F.Stangaferro, MatiasGuinebretiere, MaryseStankus, TonyGuldbrandtsen, BerntStanley, ChristinaGunia, MelanieStanley, DanaGunnarson, StefanStannard, HayleyGuo, AizhenStar, LauraGuo, KaijunStarzyński, Rafał R.Guo, Mei-JinŠťastník, OndřejGuo, ShuangshuangStayton, TristanGuo, WenjingStear, MichaelGupfinger, ReinhardStefanetti, ValentinaGupta, KuldeepStefaniak, TadeuszGuralp, HilalStefaniuk-Szmukier, MonikaGurgul, ArturStein, AndrewGurung, Nar K.Steinberg, Eliana RuthGustison, Morgan L.Steinhagen, DieterGutiérrez, Ana MaríaStejskal, VlastimilGutierrez, JuanStella, AlessandraGutiérrez, Raúl SánchezStella, JudithGutierrez-Blanco, EduardoStellato, AnastasiaGuy, JonathanStelletta, CalogeroGuzhva, OleksiyStephan, WildeusGuzmán-Pino, SergioStephens, TanyaHa, James C.Stępniowska, AnnaHabig, ChristinSter, CélineHącia, EwaSteuer, Ashley E.Hać-Szymańczuk, ElżbietaStocco, GiorgiaHadley, PhilStock, EmmelieHagen, LiveStojak, JoannaHagiya, KoichiStojkov, JaneHaigh, AmyStone, AmandaHairgrove, ThomasSt-Pierre, BenoitHalagarda, MichałStradaioli, GiuseppeHalas, VeronikaStrand, EricHalbrook, Richard S.Strandberg, ErlingHales, JanniStranieri, AngelicaHall, CarolStrappini, AnaHall, Edward J.Stratmann, ArianeHall, Emily J.Streck, André FelipeHall, KatieStrillacci, Maria GiuseppinaHall, Nathaniel J.Štritof, ZrinkaHall, RobynStrive, TanjaHall, SarahStrychalski, JanuszHalstead, BrianStrydom, Phillip E.Hamann, SteffenStuart, BryanHamilton, PatrickStumpff, FriederikeHammerl, Jens AndreSturaro, EnricoHamonic, GlennSturgill, Tracy L.Hampton, JordanStygar, AnnaHan, QianSu, GuoshengHan, XuefengSu, HuaweiHandl, StefanieSu, ShuoHandler, JohannesSu, YongHandorf, OliverSuarez Alonso, Maria Del PilarHang, SuqinSuarez-Trujillo, AridanyHankel, JuliaSuchocki, TomaszHanna, Lauren HulsmanSuchomel, JosefHanne, HansenSudhagar, ArunHannisdal, RitaSueur, CédricHanon, Jean-BaptisteSugaya, TakumaHansen, Henning OtteSugita, HaruoHansen, Mark F.Sugiyama, FumihiroHansen, Tia G. B.Sugiyama, ToshieHanson, Jeanette M.Sukhodolskaya, RaisaHanuš, OtoSukumaran, Anuraj TheradiyilHarcourt-Brown, FrancesSullivan, RyanHare, James F.Sultan, KhaledHarrison, AdrianSultana, HalimaHarrison, SabineSummer, AndreaHarrison, TaraSumner, Rebecca NicoleHart, BenjaminSun, HaiyanHart, Lynette A.Sun, LvhuiHart, SteveSun, PengHartmann, SabineSun, XiaolunHartstone-Rose, AdamSun, XuezhaoHarvey, Andrea M.Sundararajan, RajiHarvey, John W.Sunde, JanHarvey, NaomiSurai, Peter F.Hasan, Shah M.K.Susi, PetriHaskell, MarieSutherland, MhairiHassanat, FadiSuzuki, HiroshiHässig, MichaelSuzuki, ReijiHatcher, SueSuzuki, YutakaHattori, KaoruSvoboda, MartinHaussler, KevinSwalve, Hermann H.Hawes, PhilippaSwanson, JanetHawes, SloaneSwanson, KendallHayashi, Mariko KatoSwarge, Bhagyashree N.Hayashi, ShinichiroSwartz, Steven ZacharyHazel, Susan J.Swartz, Turner H.He, ChengSwelum, Ayman A.Heath, SarahSwerczek, Thomas W.Hedfalk, KristinaŚwiątkiewicz, MałgorzataHedman, HaydenSwinbourne, Alyce M.Heerkens, JasperSwinker, Ann M.Heidor, RenatoSwinnen, KristijnHeilen, Laura B.Świtoński, MarekHein, NilsSyahputra, KhairulHeinemann, Marcos BryanSymons, JenniferHeinicke, JuliaSzablewski, TomaszHeizmann, VeronikaSzacawa, EwelinaHejdysz, MarcinSzachniuk, MartaHejna, MonikaSzczepanek, JoannaHeld, SuzanneSzczepankiewicz, BarbaraHeleski, CamieSzczerba-Turek, AnnaHeller, E. DanSzczurek, WitoldHellmen, EvaSzeleszczuk, ŁukaszHemingway, AnnSzendrő, KatalinHempel, SabrinaSzendrő, ZsoltHempstead, MelissaSzentiványi, TamaraHemsworth, Lauren M.Szewczuk, MałgorzataHenao, SantiagoSzóstek-Mioduchowska, AnnaHenao-Díaz, Yuly AlexandraSzteyn, JoannaHendricks, William P. D.Szumacher-Strabel, MalgorzataHendrickson, DeanSzymańska-Czerwińska, MonikaHenkanaththegedara, SujanSzyndler-Nędza, MagdalenaHenklewska, MartaTabata, RyoichiHennessy, DeirdreTabor, GillianHennessy, MichaelTaciak, MarcinHeo, Jung MinTaddei, SimoneHeppenheimer, ElizabethTadich, TamaraHeras-Molina, AnaTagang, AluwongHerborn, KatherineTagliapietra, FrancoHerbut, PiotrTaguchi, ToruHermansen, John ErikTahamtani, Fernanda M.Hernandez Medrano, JuanTajchman, KatarzynaHernández Pérez, Maria Del PilarTajima, MotoshiHernández, Felipe HernándezTajima, YukoHernández, Joaquín R.Takagi, YasuakiHernandez-Castellano, Lorenzo E.Takahashi, HideakiHernandez-Gomez, ObedTakahashi-Omoe, HiromiHernot, SophieTakaya, TomohideHeroldová, MartaTakehiro, NishidaHerrelko, ElizabethTakeshi, TsukaHerrera Haro, José GuadalupeTalamini, EdsonHerrera, MarcelinoTalenti, AndreaHerrick, Jason R.Tallo-Parra, OriolHerskin, Mette S.Talukder, SaranikaHervás, GonzaloTambella, Adolfo MariaHervera, MartaTamburro, RobertoHerwig, EugeniaTan, BieHerzog, CatherineTan, QiHerzog, HaroldTan, ZhiliangHess, ClaudiaTang, KaiHessle, AnnaTang, YulongHeyrman, EvertTang, ZhaoxinHibino, YusukeTang, ZhonglinHickman, DebraTaniguchi, MasaakiHickson, RebeccaTantillo, Giuseppina MariliaHidalgo, Carlos O.Tanui, Collins K.Highland, Margaret A.Tao, FeifeiHiguera, Cristina SaroTaponen, SuviHiki, KyoshiroTarantola, MartinaHildebrand, JoannaTardella, Federico MariaHildebrandt, NicolaiTarr, GarthHildreth, BlakeTarradas Font, JoanHildreth, EasonTarricone, SimonaHilgerloh, GudrunTassone, SoniaHill, FraserTavares-Dias, MarcosHill, HeatherTaylor, LucyHiller, ThomasTaylor-Bowden, ThyneiceHillmann, EdnaTe Pas, MarinusHinch, GeoffTedds, HelenHinde, KatieTeerds, Katja J.Hiney, KrisTejedor, María TeresaHinkle, Nancy C.Telezhenko, EvgenijHinney, BarbaraTell, LisaHirayasu, HirofumiTella, José L.Hirofumi, NogamiTellez-Isaias, GuillermoHirsbrunner, GabrielaTellez-Lance, AngelaHnida, John A.Teng, XiaohuaHoagstrom, ChristopherTerčič, DušanHoareau, ThierryTeresa, Pacchioli MariaHobday, AlistairTerler, GeorgHoberg, Eric P.Terlouw, E.M. ClaudiaHockenhull, JoTernman, EmmaHockenhull, JoannaTerrazas, Luis I.Hodgkinson, SuzanneTerré, MartaHoelzle, Ludwig E.Terrill, ThomasHoenig, MargaretheTerryn, SanneHof, AnouschkaTesi, MatteoHoff, StevenTetens, JensHoffman, MariaTettamanti, GianlucaHogg, RachelThakur, Krishna KumarHolinger, MirjamTheuerkauf, JörnHollis, Anna R.Thibodeau, AlexandreHolst, Bodil StrömThielke, Lauren E.Holzhauer, MennoThiemann, Alexandra K.Honaker, Christa F.Thoene, MelissaHonda, MasahikoTholen, ErnstHong, Bo-YoungThomareis, ApostolosHong, Cheng-YihThomas, JulieHong, JinsuThompson, LoganHong, Yeong HoThomson, JackHonneffer, Julia B.Thomson, JenniferHood, JenniferThomson, Peter C.Hoole, DavidThomspon, KimHooper, DanielThöne-Reineke, ChristaHopper, LydiaThonney, MichaelHoradagoda, NeilThornton-Kurth, KaraHorák, PetrThymann, ThomasHorback, KristinaTian, ChangxuHorin, PetrTian, QiyuHornick, Jean-LucTickel, JimmyHornung, BastianTiemann, IngaHorohov, David W.Tietze, ThomasHorowitz, AlexandraTiezzi, FrancescoHorsburgh, AnnTilocca, BrunoHorst, EgonTinker, JulietteHorvat, SimonTinkler, StacyHosoe, MisaTiplady, CatherineHosoyamada, MakotoTita, OvidiuHossain, Md. SakhawatTizzani, PaoloHostens, MielTkaczynski, PatrickHotea, IonelaToaff-Rosenstein, Rachel L.Hotos, George N.Todaro, MassimoHötzel, MariaTofastrud, MortenHou, FujiangToftaker, IngridHoue, HansTokach, MikeHoupt, Katherine AlbroTokarska, MałgorzataHouse, JohnTokmina-Lukaszewska, MonikaHovinen, MariTolhurst, Bryony A.Howard, Tanya M.Tomasevic, IgorHowell, TiffaniTomassone, LauraHoy, SteffenTomaszewska, EwaHsiao, Chung-DerTomaszewska-Zaremba, DorotaHsieh, KuangwenTomczyk, ŁukaszHsieh, Ming KunTomczyk-Wrona, IwonaHsu, Jih-TayTomonaga, ShozoHsu, Te-HuaToms, DerekHu, JianhongTong, ReinaHu, JielunTonizza De Carvalho, Nelcio AntonioHu, NianZeToomer, OndullaHu, XiaoxiangTopic-Popovic, NatalijaHu, ZhiyongTorren, MargalidaHua, GangTorres, AlexandrHua, GuohuaTorres, João PauloHuang, FeiruoTorres, Rita TinocoHuang, Li-TungTorres-Acosta, Juan FelipeHuang, LvwenTorsteinsdottir, SigurbjorgHuang, San-YuanTorti, ValeriaHuang, YongzhenTóth, TamásHuber, NikolausTouchard, Arturo GarcíaHuck, MarenTous, NuriaHuerlimann, RogerTovar-Ramírez, DarielHuertas, StellaToyomizu, MasaakiHughes, CourtneyTrabalza-Marinucci, MassimoHughes, KrisTrachsel, MaryHuh, Jae-WonTrammell, SamuelHuizing, XanderTrauffler, LindaHułas-Stasiak, MonikaTraulsen, ImkeHulbert, LindseyTråvén, MadeleineHultgren, JanTravis, Holly J.Hulva, PavelTremolada, GiovanniHuman, StaceyTremoleda, Jordi L.Humer, ElkeTretola, MarcoHumm, KarenTrijillo-Gonzales, AlejandroHumphreys, JamesTrimborn, ManfredHund, AlexandraTroan, BrigidHunt, HayleyTrocino, AngelaHunt, JodeeTroedsson, Mats H.T.Huntingford, JaniceTrombetta, Maria FedericaHuo, Bing-XingTrusch, FranziskaHuo, LijunTryjanowski, PiotrHussain, HidayatTsakmakidis, Ioannis A.Hussein, MaythamTsang, Susan M.Husvéth, FerencTsantarliotou, Maria P.Hutson, KateTsiouris, VasiliosHutz, ReinholdTsiplakou, EleniHwang, In-SulTsoi, StephenHyde, RobertTsuji, YamatoIaffaldano, NicolaiaTsukahara, TakamitsuIannetti, LuigiTsuruta, ShogoIanni, AndreaTsutsui, ShigeyukiIaria, CarmeloTu, YanIbanescu, IulianTually, SelinaIbáñez, Rodrigo A.Tudisco, RaffaellaIben, ChristineTufarelli, VincenzoIbrahim, FandiTung, Tao-HsinIchihashi, HidekiTunick, MichaelIchinohe, ToshiyoshiTurcios, Ariel E.Icyuz, MertTurini, LucaIgawa, TakeshiTurk, RomanaIguchi, TaisenTurner, Patricia V.Ihara, MasaruTurner-Walker, GordonIllera, Juan CarlosTurpin-Nolan, SarahImperatore, RobertaTuśnio, AnnaIndugu, NagarajuTyers, AlexandraInfascelli, FedericoTylan, CatherineIngala, Melissa R.Tynes, ValarieIngham, Aaron B.Uberoi, AayushiIngle, HarshadUccheddu, StefaniaInglis, G. DouglasUdala, JanIngwell, Laura L.Uddin, Muhammad JasimInic-Kanada, AleksandraUebanso, TakashiIniesta-Cuerda, MaríaUnchwaniwala, NuruddinInnis, CharlesUngerfeld, Emilio M.Iqbal, YasirUpadhyay, MaulikIrimajiri, MamiUpjohn, MelissaIrons, PeteUrban, TomasIrvine, LeslieUrban-Chmiel, RenataIsakova-Sivak, IrinaUwiera, RichardIshfaq, MuhammadUyeno, YutakaIshihara, KenjiVäärikkälä, SofiaIshikawa, AkiraVacher, Jean-PierreIslam, Md ZohorulVaglio, StefanoIslam, ZohorulVaiman, DanielIson, Sarah H.Valaja, JarmoIso-Touru, TerhiValasi, IreneIstván, NagyValastro, CarmelaItze-Mayrhofer, CorinaValcácia Silva, SildivaneIvankovic, AnteValdés, CarmenIvemeyer, SilviaValdés-Baizabal, CatalinaIwasaki, TomohitoValdez, JoseIwasaki, WataruValente, Bruno DouradoIwu, ChinweValente, Fabrício LucianiIyer, ChitraValenti, BernardoIzquierdo, MercedesValentini, AlessioJackson, JohnValentini, SimonaJacob, JacquelineValenzuela, CarolinaJacobs, LeonieValero, YulemaJacobsen, MonicaValgaeren, BonnieJacquiet, PhilippeVallée, EmilieJadhav, ShyamalagauriValsecchi, Paola MariaJagannathan, VidhyaVan Amburgh, MichaelJagga, SupriyaVan De Vis, HansJakubczak, AndrzejVan Den Boom, RobinJalali, Beenu MozaVan Den Brand, HenryJalongo, Mary RenckVan Den Esker, Marielle H.James, CharlotteVan Der Aar, PietJanas, AdamVan Der Horst, GerhardJanczak, Andrew M.Van Der Pol, Carla W.Janeczek, MaciejVan Der Saag, DominiqueJaneczko, Stephanievan Dierendonck, MachteldJang, Jae-CheolVan Eerdenburg, FrankJang, Young DalVan Erp-van Der Kooij, LennyJanikowska, GrażynaVan Heezik, YolandaJaniszewski, PawełVan Hensbergen, HubertusJankovská, IvanaVan Kempen, Theo A.T.G.Jankowski, JanVan Kesteren, FreyaJanssens, GeertVan Loon, Johannes P.A.M.Janssens, StevenVan Marle-Köster, EsteJanuś, EwaVan Niekerk, TheaJanzen, EugeneVan Opzeeland, Ilse C.Jarosz, ŁukaszVan Parys, AlexanderJavǔrková, Veronika GvoždíkováVan Soom, AnnJawor, Paulinavan Staaveren, NienkeJaworski, NeilVan Steenbeek, Frank GeurtJayawardena, DulariVan Vuuren, BettineJażdżewska, IwonaVan Winden, StevenJefferson, Wendy N.Vanderperren, KatrienJekl, VladimírVangen, OddJelinski, MurrayVanlierde, AmelieJenckel, MariaVannucchi, Camila InfantosiJenkins, Thomas C.Vanore, MariaJennings, Rebecca L.Vanselow, JensJensen, KirstyVarga, CsabaJensen, Ole T.Varga, MátéJensen, ThomasVarga, Zoltan M.Jenson, IanVargas-Bello-Pérez, EinarJenssen, HåvardVarga-Visi, EvaJenvey, CaitlinVarona, LuisJerez, NancyVasconcellos, Ricardo SouzaJerez, SalvadorVasconcelos, Ana Carina NogueiraJerez-Cepa, IsmaelVasconcelos, Elton J. R.Jerônimo, GabrielaVasdal, GuroJewell, Dennis E.Vasileiou, Natalia G.C.Jewgenow, KatarinaVastolo, AlessandroJeyanathan, JeyamalarVaughan, MelJeyaruban, GilbertVaughn, Mathew.A.Jeżewska-Frąckowiak, JoannaVaux, FelixJia, BeibeiVázquez Bringas, Francisco JoséJiang, Jian-PingVecchiato, Carla GiudittaJiang, ShijinVeerus, LiisaJiang, ShouqunVega-Pla, Jose LouisJiang, SiwenVegarud, Gerd ElizabethJiang, XunpingVeiga Dos Santos, MarcosJiang, YunVeiga, InêsJin, MinVeldkamp, TeunJiří, PatokaVéliz Deras, Francisco GerardoJobling, MalcolmVenâncio, CátiaJoblon, MelissaVenâncio, Émerson JoséJohansen, Matt D.Vengušt, ModestJohnson, Aime K.Venkatesan, Jagadeesh K.Johnson, AmyVentrella, DomenicoJohnson, Dylan M.Vera Ávila, Héctor RaymundoJohnson, PaulVerardo, Lucas LimaJohnson, SallyVerbrugghe, AdronieJohnston, LeeVerdon, MeganJohson, LynellVergneau-Grosset, ClaireJokimäki, JukkaVerleih, MariekeJonas, KimVermeer, Herman MaartenJonckers, ElisabethVerri, Waldiceu A.Jones, DarrylVerrinder, JoyJones, ElisabethVerruck, SilvaniJones, PhilipVerstegen, John P.Jones-Diette, Julie S.Verstraete, FrankJongman, EllenVetrano, StefaniaJordan, BrianViana, DavidJorge, ErikaViana, FlaviaJosa, Laia BlaviViana, Gabriel Da SilvaJosé Manuel, Perea MuñozViana, Joao Henrique MoreiraJoshi, ChetanchandraVibart, RonaldoJourdain, ElsaVicente, FernandoJoy, MargalidaViciano Badal, JoanJózefiak, AgataVicino, Greg A.Juárez, ManuelViegas, CarlosJudycka, SylwiaVieira, Bruno SerpaJulliand, VéroniqueVieira, Felipe Do NascimentoJulliard, RomainVieira, RicardoJuodka, RobertasVielma, JouniJurado, Macarena M.Vigors, BelindaJuškienė, VioletaVigors, StaffordJuste, RamónVikram, AmitJůzl, MiroslavVilar Ares, Maria J.Kaba, JaroslawVilla, LucaKabir, MorvaridVillagomez, DanielKačániová, MiroslavaVillagrá García, ArantxaKaczmarek, SebastianVillanova, Fabiola ElizabethKaeffer, BertrandVillarruel-López, AngélicaKagawa, NaoViñas, MiguelKaji, KoichiVincent, AvivaKakinuma, MikiVincenti, LeilaKakoi, HironagaVincēviča-Gaile, ZaneKakuda, TsutomuVinciguerra, Maria TeresaKalaitzakis, EmmanouilVingo, Paolo DeKaldhusdal, MagneVioleta, RazmaitėKalus, KajetanVisscher, ChristianKandasamy, RamVisser, CarinaKaneda, MasahiroVisser, KathalijneKaneko, GenVitale, Kristyn R.Kaneniwa, MasakiVitale, MaríaKang, HuiminVitali, MarikaKang, JinheViuda-Martos, ManuelKang, SeongVivarelli, FabioKapica, MałgorzataVizcaíno, Antonio JesúsKapusta, AndrzejVizzarri, FrancescoKaraca, SerhatVogeler, Colette S.Karadjian, GregoryVogl, ClausKaramon, JacekVoigt, KatjaKaranis, PanagiotisVolfova, MartinaKaratzia, Maria-AnastasiaVolsche, ShellyKaravoltsos, SotiriosVon Essen, EricaKarcher, DarrinVon Eugen, KayaKarenina, KarinaVon Keyserlingk, MarinaKarlsson, ErikVon May, RudolfKarpiński, MirosławVon Samson-Himmelstjerna, GeorgKasapidou, EleniVonk, JenniferKasarda, RadovanVoslářová, EvaKasbaoui, NaïmaVranken, ErikKasimanickam, Vanmathy R.Vrentas, Catherine E.Kasperek, KornelVyas, DiwakarKasper-Völkl, ClaudiaWaal, Theo DeKasprowicz-Potocka, MałgorzataWachirapakorn, ChalongKasprzyk, AnnaWade, ClaireKass, MarkoWagener, KarenKatharina, HohlbaumWagle, Basanta RajKatsafadou, AngelikiWagman, Jeffrey B.Katz, Aron D.Wagner, BrooklynKaukonen, EijaWagner, LianeKaushik, PrashantWähner, MartinKaushik, SwatiWai, LowKawamura, HiroakiWaite, KarenKawasaki, KiyonoriWakshlag, Joseph J.Kawashima, ChihoWalker, JenniferKawata, YukichikaWalker, Rebecca L.Kawęcka, AldonaWall, HelenaKearton, TellisaWallenbeck, AnnaKeates, HelenWallner, BarbaraKędzierski, WitoldWalshe, NicolaKeegan, JasonWalter, Jasmine A.Keel, BrittneyWalter, MelanieKeele, John W.Walugembe, MuhammedKehoe, SylviaWandesforde-Smith, GeoffreyKeim, Juan PabloWang, BingKeisuke, KoyamaWang, CunwenKeleher, Madeline RoseWang, DingKells, NikkiWang, FangKelton, David F.Wang, FenglaiKemenesi, GáborWang, GangKemp, Peter DavidWang, HaifengKemper, NicoleWang, Hao-ChingKenéz, ÁkosWang, HongrongKennedy, UttaraWang, JiakunKenyon, Paul R.Wang, JiaqiKeogh, KateWang, JingKerr, Caroline AudreyWang, JingjingKessler, SharonWang, JiwenKeuling, OliverWang, JunjunKhalid, HattafWang, KaiKhamas, WaelWang, LifangKhan, AjmalWang, MengzhiKhan, RajwaliWang, QishanKhattak, FarinaWang, Sheng-yiKhazandi, ManouchehrWang, Shu-YinKhezri, AbdolrahmanWang, SongboKholová, IvanaWang, WenceKhorozyan, IgorWang, XiKhurshid, ChrowWang, XiangruKiaei, MahmoudWang, XiaofanKiarie, Elijah G.Wang, XiaojunKicińska, AlicjaWang, XiuqiKiczorowska, BożenaWang, Xu (Auburn)Kiddie, JennaWang, Xu (Wuhan)Kiełbowicz, ZdzisławWang, YachunKielland, CamillaWang, YuxiKienapfel, KathrinWang, ZaisenKierończyk, BartoszWard, AlisonKieson, EmilyWard, Samantha J.Kiezun, MartaWark, Jason D.Kikuchi, KiyoshiWarner, LesleyKikusato, MotoiWarren, Lori K.Kilcawley, KieranWarren, PhilipKiley-Worthington, MartheWarwick, CliffordKilroy, DavidWarzych, EwelinaKim, Beob GyunWascher, Claudia A. F.Kim, Choon YoungWashburn, SteveKim, Dal YoungWasylishen, AmandaKim, Eun JoongWaszkiewicz, EwaKim, Haeng-hoonWatanabe, GenKim, Hyeon TaeWaterhouse, TonyKim, Hyeon-Joong AidenWatkins, Peter J.Kim, Hyeun BumWatson, Aaron M.Kim, InhoWauters, Lucas A.Kim, Jong GugWazed, Md AbdulKim, JunmoWealleans, AlexandraKim, LouWeary, DanKim, MinseokWeaver, AliceKim, Myung-HooWebb, Edward C.Kim, Raymond HWebb, Jonathan L.Kim, Seong-GiWebb, Molly A.H.Kim, SeonghunWebber, Sarah EllenKim, SungwonWebster, JohnKim, WooWeese, ScottKim, WootaeWei, CaihongKind, Karen L.Weiler, UlrikeKing, MeaganWeimer, PaulKing, Sarah R.B.Weimer, Shawna L.Kingan, MichaelWeiss, MartinaKinoshita, MasatoWeller, Joel IraKirchner, Marlene KatharinaWels, HarryKirczuk, LucynaWen, XiaoboKirkeby, CarstenWenk, CasparKiser, Jennifer N.Werne, SteffenKishigami, SatoshiWerner, DanielaKishimoto, JiroWerner, MarianneKitchener, AndrewWerth, Alexander J.Kitsiouli, EiriniWestreicher-Kristen, EdwinKittelsen, Käthe EliseWhitaker, Brian D.Kittler, SophieWhite, John KerrKjos, Nils PetterWhite, LaurenKlaoudatos, DimitriosWhite, Nathaniel A.Klaumann, FranciniWhite, PeterKlebaniuk, RenataWhite, RehemaKlećkowska-Nawrot, JoannaWhite, Stephen DavidKlee, SaschaWhitehouse, NancyKlein, Dylan J.Whitehouse-Tedd, KatherineKlein, Joshua D.Whitfield, Steven M.Klein, WilfriedWhiting, TerryKlement, EyalWhittaker, AlexandraKlich, DanielWichert, BrigittaKlockiewicz, MaciejWick, MacdonaldKloesch, GerhardWickens, CarissaKlop-Toker, KayaWickersham, TryonKlos, MatthewWicksten, MaryKlunemann, MartinaWidowski, Tina MKlymiuk, Michele ChristianWieczorek, KingaKnaga, SebastianWieland, MatthiasKnapczyk-Stwora, KatarzynaWiener, DominiqueKnauer, WhitneyWiener, PamKnierim, UteWierzbowska, IzabelaKnight, AndrewWigham, Eleanor E.Knight, PeterWigham, EllieKnobel, DarrynWijers, MatthewKnottenbelt, DerekWiktor, ArturKoblmüller, StephanWilk, IzabelaKoch, ChristianWilkes, EdwinaKoch, Daniel A.Wilkins, HeatherKoch, FranziskaWilliams, ByronKochan, JoannaWilliams, Charlotte AmdiKochanowski, MaciejWilliams, EllenKodama, DaisukeWilliams, HowelKoehler, GeoffWilliams, Jane M.Kofler, JohannWilliams, Larissa M.Kogan, LoriWilliams, RoderickKogut, Michael H.Williams, SusanKohda, TakashiWillis, Theodore V.Kohlmann, KlausWills, AlisonKokkonen, TuomoWilson, GeorgeKokokiris, LambrosWilson, Helen F.Kokoszyński, DariuszWilson, JuliaKokou, FotiniWilson, MeganKolakshyapati, ManishaWimbush, KarenKolasa-Wołosiuk, AgnieszkaWindisch, WilhelmKolbe, ThomasWiniarska-Mieczan, AnnaKoledova, ZuzanaWin-Shwe, Tin-TinKolenda, MagdalenaWintle, BrendanKoliesnik, IevgenWisselink, HenkKolisko, MartinWiszniewska-Łaszczych, AgnieszkaKołodziejski, PawełWitkowska-Piłaszewicz, OlgaKoltes, DawnWnuk, AgnieszkaKomaki, ShoheiWnuk-Pawlak, ElżbietaKominakis, AntonisWohlers, JeniferKomissarov, AlekseyWoinarski, JohnKomiyama, TomoyoshiWójcik, AnnaKomlósi, IstvánWójcik, MaciejKomosa, MarcinWojdak-Maksymiec, KatarzynaKoncicki, AndrzejWojtaś, JustynaKong, ChangsuWojtulewicz, KarolinaKong, Il-KeunWojtysiak, DorotaKong, XiangfengWolfensohn, SarahKongsted, Anne GreteWolny-Koładka, KatarzynaKonieczka, PawełWong, Marty Kwok-ShingKonold, TimmWood, BenKonstantakopoulou, FoteiniWood, JeffKopp, StevenWood, KevinKopper, Jamie J.Wood, MartynKorczyński, MariuszWoodbury, BryanKordowitzki, PawełWoods, Jocelyn M.Korsgaard, Trine StokbroWoodward, ElizabethKorwin-Kossakowska, AgnieszkaWoodworth, Jason C.Korzekwa, Anna J.Wormald, DennisKos, IvicaWotton, StephenKosacka, JoannaWoźniakowski, GrzegorzKosan, ChristianWright, PatrickKoseniuk, AnnaWróbel, BarbaraKosova, KlaraWróblewska, BarbaraKostomitsopoulos, Nikolaos G.Wronski, TorstenKostoulas, PolychronisWu, ShengruKotrschal, KurtWu, ShugengKotula-Balak, MałgorzataWu, WangjunKougioumtzis, AlexandrosWu, XinKoutoulis, KonstantinosWu, XinshengKoutsos, LizWu, ZhonghongKowal, KrzysztofWurtz, Kaitlin E.Kowalczuk-Vasilev, EdytaWürtz, SvenKowalczyk, AlicjaWutke, SaskiaKowalczyk, ArturWuyts, SanderKowalewska-Łuczak, IngaWyatt, TanyaKowalska, AgataWydooghe, ElineKowalska, DorotaWyffels, SamuelKowalski, Radoslaw KajetanWysok, BeataKowtharapu, Bhavani S.Wysokińska, AnnaKozdrowski, RolandXia, ChengKozdru, WojciechXiao, YangKozloski, GilbertoXiaojun, YangKrakowski, LeszekXiccato, GerolamoKralik, ZlataXie, QingmeiKramer, Laura HelenXie, ZiliKrasheninnikova, AnastasiaXiong, BenhaiKrasnov, AlekseiXu, DongdongKrauel, Jennifer J.Xu, JieKrauze, MagdalenaXu, JingjingKrauze-Gryz, DagnyXu, LingyangKrebs, Bethany L.Xu, XiaoKress, KevinXue, TingKreuzer-Redmer, SusanneYakobson, Boris A.Krieg, JochenYamamoto, TakeshiKritchevsky, Janice E.Yamamoto, YojiKřížová, LudmilaYamanaka, SatoshiKrizsan, Sophie J.Yamanashi, YumiKrol, BarbaraYamane, AkihiroKról, JolantaYamano, KeisukeKróliczewska, BożenaYamauchi, HiroyukiKrömker, VolkerYamayoshi, SeiyaKrommweh, Manuel S.Yan, PingKruszynski, WojciechYan, QingpiKrzanowski, RomanYang, Cheng-YaoKrzysztof, AnuszYang, HuanshengKrzywonos, MałgorzataYang, InhoKubasiewicz, Laura M.Yang, LingKubelková, PetraYang, QienKubiak, KatarzynaYang, QingxiangKubinyi, EnikoYang, QiyuanKubota, MasatoshiYang, Run-junKucharska, KarolinaYang, ShulinKuczyńska, BeataYang, Shyi-KuenKuda, OndrejYang, Wei-hsiungKues, Wilfried A.Yang, WenzhuKuhnke, SandraYang, WucaiKulick, DonYang, XuefenKulkarni, RaviYang, YongxinKulma, MartinYang, ZhangpingKulshreshtha, GarimaYao, GuolinKumar, GokhleshYao, JunhuKumar, PraveenYao, WenKumar, RajeshYasuda, MasahiroKupczyński, RobertYasuike, MotoshigeKurita, JunYayota, MasatoKurowicka, BeataYazdani, MaziarKusama, KazuyaYeste, MarcKusano, KanichiYin, JieKuzniar, AliceYin, JingdongKvist, LauraYin, RenfuKwiecień, MałgorzataYin, TongKwon, Young M.Yin, WenqingKyriakakis, JoannaYngvesson, JennyLaarman, AnneYokoi, HayatoLabroussaa, FabienYokoyama, SaichiroLacham-Kaplan, OrlyYon, LisaLacitignola, LucaYonekura, ShinichiLackner, ReinhardYoo, Han SangLacroix, MatthieuYoon, HachungLadewig, JanYoon, MinjoongLadlow, JaneYosef, ReuvenLadwig, MechthildYoshinaga, JunLafrancois, TobenYou, XinxinLaguna Conceição Meirelles, SarahYoung, Jette F.Lahbib-Mansais, YvetteYounis, ShadyLai, OlimpiaYravedra, JoséLaliotis, George P.Yu, BINGLamagna, BarbaraYu, BoLamas, María IsabelYu, Il-JeoungLamas-Toranzo, IsmaelYu, MeiLamb, DavidYu, QingLambert, MarkYu, Yu-HsiangLambertini, CarlottaYuan, ChunhongLamont, SusanYuan, JianminLan, XianyongYue, XiangpengLan, Yi-ChenYúfera, ManuelLancioni, HoviragYun, Hyung-MunLandete-Castillejos, TomásYun, SiningLandi, VincenzoZachut, MayaLandim- Alvarenga, Fernanda Da CruzZachwieja, AndrzejLandsberg, Gary M.Zahan, MariusLangendijk, PieterZahmel, JenniferLangeveld, JanZahoor, MuhammadLanza, MassimilianoZaǐtsev, Sergei YuLapierre, LisetteŻakowska-Biemans, SylwiaLara, Leonardo José CamargosZaleski, HalinaLardner, Herbert BartZamaratskaia, GaliaLares, FernandoZambonelli, PaoloLari, EbrahimZampiga, MarcoLaricchiuta, PietroZanella, FabianLarrondo, CristianZanella, RicardoLarzul, CatherineZanetti, Marcus AntonioLasagna, EmilianoZaniboni, LuisaLaskowski, WacławZanini, SilviaLatoch, AgnieszkaZappaterra, MartinaLatorre, Maria AngelesZarkada, AnnaLau, QuintinŻarski, DanielLaudadio, VitoZatelli, AndreaLaurà, RosariaZatterale, FedericaLaurence, MichaelZaworska-Zakrzewska, AnitaLauriault, LeonardZdenek, ChristinaLavelle, MichaelZduńczyk, SławomirLaven, LindaZdunczyk, ZenonLaven, RichardZduńczyk, ZenonLavon, YanivZecconi, AlfonsoLavrenčič, AndrejZeineldin, MohamedLawrence, Catherine E.Zeitz, JohannaLazado, CarloZelena, DoraLázaro, Victoria LuñoZelli, RiccardoLazarowski, LuciaZeng, Chang-JunLazzari, RafaelZeng, QiufengLe Cam, LaurentZeng, XiangyanLe Gall, TonyZenga, MariangelaLe, Anh V.Zerani, MassimoLe, Hieu HuuZhalniarovich, YauheniLea, RichardZhan, BinLeal Yepes, Francisco A.Zhang, FanLeal, Cláudia L.V.Zhang, GenxiLearmonth, Mark JamesZhang, GuanshiLeavesley, DavidZhang, HaijunLebelt, DirkZhang, HaoLechevalier, ValerieZhang, HongpingLeclère, MathildeZhang, HuanminLedwoń, AleksandraZhang, HuizhenLee, Chang HoonZhang, JileiLee, DeukhwanZhang, JunminLee, Hong-GuZhang, KeyingLee, Jeong SangZhang, Lu-PeiLee, Jong KooZhang, MichaelLee, Jung-WookZhang, NaifengLee, KeehoonZhang, ShihaiLee, KippeumZhang, WeiLee, Kyeong-JunZhang, XiaoyuLee, Kyung-WooZhang, YanmingLee, Phyllis C.Zhang, YingJunLee, Sang-SukZhang, ZheLee, Su A.Zhang, ZhigangLee, TaekZhao, ChunjiangLee, Tzu-TaiZhao, HongfengLeeds, AustinZhao, JianjunLeenaars, CathalijnZhao, ShengguoLees, Angela M.Zhao, WenxiuLees, John J.Zhao, XingboLéguillette, RenaudZhao, YiguangLehman, SarahZheng, AijuanLehner, StefanieZheng, WeichaoLehnert, Sigrid A.Zhong, RongzhenLehr, EdgarZhou, BoLei, ChuzhaoZhou, HuitongLeinonen, HenriZhou, Xiao-QiuLeite, Fábio Pereira LeivasZhou, XihongLeitner, GabrielZhou, YangLemiere, StephaneZhou, YanminLemme, AndreasZhu, JerryLeón Jurado, José ManuelZhu, MengjinLeonard, Brian C.Zhu, SixiLeonardo, LeonardiZhu-Barker, XiaLepczyński, AdamZi, XuejuanLeplat, JohannZięba, GrzegorzLerner, HenrikZiegler, AmandaLeroy, GregoireZielak-Steciwko, AnnaLeso, LorenzoZielińska, EwelinaLessard, MartinZilic, ZeljkoLessire, FrançoiseZimazile Sibanda, TerenceLestingi, AntoniaZimmerman, Patrick H.Letko, AnnaZinovieva, NataliaLétourneau-Montminy, Marie-PierreZitek, AndreasLettini, Antonia AnnaZizzo, NicolaLevine, DavidZnamirowska, AgataLewandowicz, JacekZobel, GosiaLewczuk, DorotaZochowska-Kujawska, JoannaLewejohann, LarsZoidis, EvangelosLewis, HelenZöls, SusanneLewis, MikeZou, XiaotingLewis, RichardZsoldos, RebekaLewis, Ronald M.Zsolnai, AttilaLey, Jacqueline M.Zsuzsanna, BőszeLeyva-Jimenez, HectorZucali, MaddalenaLi, AihuaŻukowski, KacperLi, AnningZulch, HelenLi, BaomingŻulewska, JustynaLi, CongjunZullini, AldoLi, FengeZuolo, FedericoLi, FuchangZwierzchowski, LechLi, GuangyuZybailov, Boris L.Li, JiaZygner, WojciechLi, Jiakui


